# Efficacy and Safety of IDegLira in Patients with Type 2 Diabetes Mellitus: A Systematic Review and Meta-analysis of Randomized Controlled Trials

**DOI:** 10.2174/0115733998359708250822102217

**Published:** 2025-09-11

**Authors:** Lexie Bai, Delong Liu, Ning Su, Lina Zhang, Zhiyong Yang

**Affiliations:** 1 Pharmacy College, Dali University, Dali, 671000, China

**Keywords:** IDegLira, type 2 diabetes mellitus, A1c, CVD, obesity, Insulin Resistance (IR)

## Abstract

**Introduction:**

Clinical trials indicate that IDegLira is effective in treating type 2 diabetes mellitus (T2DM). This study aims to assess the efficacy and safety of IDegLira in T2DM comprehensively.

**Methods:**

To identify relevant randomized controlled trials, we searched PubMed, Embase, Cochrane Library, Web of Science, and ClinicalTrials.gov. Risk Ratios (RR) and 95% Confidence Intervals (CI) were calculated using the Mantel-Haenszel approach for dichotomous outcomes. Mean Difference (MD) and 95% CI calculated by the inverse variance approach were applied to continuous outcomes.

**Results:**

Twelve randomized controlled trials involving 7628 participants were included in this study. Compared with control groups, IDegLira has a significant hypoglycemic effect in reducing hemoglobin A1c (MD = −0.66; 95% CI [−0.85, −0.47]; *p* < 0.00001), fasting plasma glucose (MD = −0.90; 95% CI [−1.40, −0.41]; *p* = 0.0003), self-measured plasma glucose (MD = −0.82; 95% CI [−1.22, −0.42]; *p* < 0.0001) and achieving the hemoglobin A1c level of < 7.0% (RR = 1.66; 95% CI [1.44, 1.92]; *p* < 0.00001) or < 6.5% (RR = 2.13; 95% CI [1.76, 2.57]; *p* < 0.00001). IDegLira outperforms insulin in achieving the target of HbA1c < 7.0 or < 6.5% without hypoglycemia and weight gain. Besides, IDegLira did not increase the incidence of adverse events and serious adverse events.

**Discussion:**

In this section, IDegLira’s benefits on simultaneously achieving glycemic control, weight loss, and reduced hypoglycemia risk were summarized. The statistical results were carefully interpreted in conjunction with clinical concerns regarding T2DM complications, adverse effects, and cost-effectiveness differences. An expanded discussion was conducted on integrating individualized HbA1c goals as a dual endpoint, without increasing body weight or the risk of hypoglycemia. Finally, the limitations of the present study were indicated.

**Conclusion:**

IDegLira exhibits a favorable glycemic control effect and acceptable adverse effects in T2DM. Superior performance in the target glycemic control, particularly suitable for T2DM patients who do not reach the target hemoglobin A1c and have comorbid CVD or obesity.

## 1 INTRODUCTION

The International Diabetes Federation (IDF) reports that 537 million adults (aged 20-79 years) lived with diabetes worldwide in 2021, and this number is projected to rise to 643 million by 2030 [1]. Of these people, around 90-95% have type 2 diabetes mellitus [2]. Type 2 Diabetes Mellitus (T2DM) is a metabolic disease involving both genetic and environmental factors, characterized by β-cell dysfunction, Insulin Resistance (IR), and chronic inflammation [3]. The physiological disorder causes the patients’ blood sugar to fluctuate beyond the normal level, which triggers increased microvascular and macrovascular events, resulting in serious damage to multiple target organs and even death [4]. To achieve optimal results and reduce related complications, controlling glycemic levels at the target is usually necessary. Unfortunately, in clinical practice, a large number of patients do not achieve the target hemoglobin A1c (HbA1c) level, and even if they do achieve the target HbA1c, the appropriate treatment can bring potential risks, such as weight gain and hypoglycemia.

Currently, insulin is still preferred for T2DM patients, considering its effect on glycemic control and its ability to retard progression [5, 6]. However, hypoglycemia and weight gain are the major concerns of insulin therapy [7]. Glucagon-like peptide-1 receptor agonists (GLP-1 RAs) are effective agents for glycemic control. They have the benefits of protecting pancreatic β-cell function, increasing insulin sensitivity, and minimizing side effects [8]. The combination of GLP-1 RA and insulin is a reasonable medication design [8, 9]. Typically, IDegLira is a fixed-ratio combination of an insulin analogue (insulin degludec) and a GLP-1 RA (liraglutide) [10], in which insulin degludec effectively reduces Fasting Plasma Glucose (FPG). In contrast, liraglutide exerts glycemic control with a low risk of hypoglycemia and reduces appetite and body weight [11]. Therefore, IDegLira effectively bridges critical gaps in insulin therapy by combining glycemic control with weight loss and reducing the risk of hypoglycemia. In the past decade, IDegLira's therapeutic data from public reports remained limited. As a new-type combination drug, its characteristics may not be well understood by clinicians and patients with T2DM. More recently, new Randomized Controlled Trials (RCTs) and clinical reports have been published. This meta-analysis assessed the effectiveness and safety of IDegLira based on comprehensive and updated data of IDegLira in T2DM, paying special attention to target glycemic control and the composite endpoint of HbA1c.

## 2 MATERIALS AND METHODS

This study adhered to the Preferred Reporting Items for Systematic reviews and Meta-Analyses (PRISMA) guidelines [12], and the protocol for this study was registered in PROSPERO (registration no. CRD42023418973).

### 2.1 Data Sources and Search Strategy

Studies were searched using PubMed, Embase, Cochrane Library, and Web of Science from the inception of each database to April 22, 2023. Additional relevant information on studies was sourced from ClinicalTrials.gov. According to the Participants, Interventions, Comparisons, Outcomes, and Study design (PICOS) principles, an advanced search was conducted using a combination of Medical Subject Headings (MeSH) terms and related keywords. The MeSH terms included “IDegLira”, “Diabetes Mellitus, “Type 2,” and “Randomized Controlled Trial”. Only published articles in the English language were included in our study. Detailed search strategies are provided in the Supplementary Material, specifically in the Literature search section.

### 2.2 Selection and Eligibility Criteria

The search results were imported into NoteExpress management software and automatically deduplicated. Two reviewers independently screened articles at the title and abstract level. Then, eligible records were assessed comprehensively in full text according to the predetermined inclusion criteria. Any disagreements were resolved through discussion to reach a consensus or by consulting a third reviewer.

The specific eligibility criteria were established prior to the search. Inclusion criteria: (1) T2DM patients; (2) IDegLira *versus* placebo or any other antidiabetic drugs; (3) Reported data for any of the following specific effectiveness and safety outcomes: (a) change in HbA1c, (b) change in FPG, (c) change in Self-Measured Plasma Glucose (SMPG), (d) change in body weight, (e) the proportion of participants achieving the HbA1c level of < 7.0% or 6.5%, (f) the proportion of participants achieving the HbA1c level of < 7.0% or 6.5% without hypoglycemia and weight gain, (g) hypoglycemia, (h) Adverse Events (AEs), (i) and Serious Adverse Events (SAEs). Exclusion criteria: (1) non-RCTs; (2) abstracts, comments, or protocol; (3) non-fixed-dose combination; (4) treatment duration < 12 weeks; (5) animals or healthy human subjects; (6) post hoc analysis; (7) no related outcomes; (8) duplicates: For different reports of the same trial, we included the one with more related information.

### 2.3 Data Extraction

Two researchers used predesigned Excel spreadsheets to independently extract the following data from each included study: first author, publication year, National Clinical Trial (NCT) number, intervention drugs, control drugs, sample size, average age, gender ratio, HbA1c and body weight at baseline, diabetes duration, treatment duration, and the efficacy and safety outcomes. Efficacy outcomes included changes in HbA1c, FPG, SMPG, and body weight, the proportion of participants achieving an HbA1c level of < 7.0% or < 6.5%, and the proportion of participants achieving an HbA1c level of < 7.0% or < 6.5% without experiencing hypoglycemia and weight gain. Safety outcomes included hypoglycemia, AEs and SAEs. Among these outcomes, the primary outcomes were the change in HbA1c, target glycemic control, FPG, and hypoglycemia. Any discrepancies between the two data extractors were resolved through consensus or consultation with another author. Eventually, the extracted data were compiled together by the two researchers.

### 2.4 Risk of Bias Assessment

The risk of bias was assessed with the Cochrane Collaboration’s tool [13]. Two reviewers independently assessed the risk of bias based on seven domains (random sequence generation, allocation concealment, blinding of participants and personnel, blinding of outcome assessment, incomplete outcome data, selective reporting, and other bias). Ambiguous issues were resolved through discussion or consulting a third author. The risk assessment of each domain was rated as “low risk of bias”, “unclear risk of bias”, or “high risk of bias”. Overall bias risk was considered low only when the risks in all domains were also low.

### 2.5 Data Synthesis and Statistical Analysis

Statistical analysis was conducted with Review Manager 5.4 software and StataMP 17.0 software. Risk ratios and 95% confidence intervals (CIs) were calculated using the Mantel-Haenszel approach for dichotomous outcomes. In comparison, mean differences and 95% CIs were calculated using the inverse variance approach for continuous outcomes. It was considered statistically significant when the *p*- value < 0.05. Statistical heterogeneity was assessed by Tau^2^, Chi-square (χ^2^), and I-square (I^2^). *p* < 0.10 and I^2^ > 50% were considered significant heterogeneity; a random-effect model was applied; otherwise, a fixed-effect model was applied. We conducted sensitivity analysis by the “leave-one-out” method. Subgroup analysis was performed based on control group types (GLP-1 RA, insulin, placebo). Meta-regression was performed to explore the potential sources of heterogeneity further. Publication bias was tested by visual inspection of funnel plots and the LFK index of the Doi plot. When significant publication bias was detected in certain outcomes, we performed non-parametric trim and fill analysis to investigation potentially missing studies due to publication bias and test the results.

## 3 RESULTS

### 3.1 Search Results

In the initial search, 672 relevant studies were identified. We retained 335 studies after removing duplicates. At the title and abstract level, 317 studies were removed, and 18 were retained for full-text evaluation. In the remaining 18 studies, 6 studies were excluded because they did not use a fixed-dose combination (n = 2), lacked related data (n = 2), or provided duplicate data (n = 2). Finally, 12 studies were included in this systematic review and meta-analysis. The detailed selection process is shown as Fig. (**1**).

### 3.2 Study Characteristics

The main characteristics of the trials included in the study are presented in Table **1**. A total of 7628 participants were involved in 12 studies, which were RCTs published between 2014 and 2022. In the control group, one study [22] used a placebo, four studies [14, 17, 21, 24] used GLP-1 RA, and ten studies [14-20, 23-25] used insulin. Among the 12 studies, four were double-blind and eight were open-label. All 7628 participants had taken an antidiabetic drug before the trial. Background hypoglycemic therapy included oral antidiabetic agents alone or in combination with insulin. Among the studies included, four were conducted in multiple locations of China or Japan, while the remaining eight were conducted across multiple countries. There were eleven studies with an intervention duration of 26 weeks and one study with a duration of 52 weeks.

### 3.3 Risk of Bias Assessment

All studies were assessed as having a low risk of selection bias, detection bias, attrition bias, and reporting bias. Eight studies [14, 16, 17, 19-21, 23, 24] were classified as having high performance bias. All studies were sponsored by the manufacturer, which contributed to the high bias on other sources (Fig. **2**).

### 3.4 Meta-Analysis of Efficacy Outcomes

#### 3.4.1 HbA1c

All RCTs were included in the meta-analysis of HbA1c changes. Compared with control groups, IDegLira significantly reduced HbA1c (MD = −0.66; 95% CI [−0.85, −0.47]; *p* < 0.00001) (Fig. **3**). Sensitivity analysis of HbA1c confirmed the robustness of the results. Coherent results were obtained in the subgroup analysis (Fig. **S1**). In terms of publication bias, the funnel plot (Fig. **S2a**) did not show any asymmetry, but minor asymmetry in the Doi plot (Fig. **S2b** ) with the LFK index of -1.80.

#### 3.4.2 Body Weight

A total of ten RCTs reported information on body weight. From the forest plot (Fig. **S3A**), there were no significant differences in body weight between IDegLira and control drugs. Sensitivity analysis of body weight verified the robustness of the results. However, we observed different results in the subgroup analysis (Fig. **S3B**). IDegLira has a significant body weight loss compared with insulin (MD = −2.05; 95% CI [−2.67, −1.44]; *p* < 0.00001), but a body weight gain compared with GLP-1 RA (MD = 2.97; 95% CI [2.34, 3.59]; *p* < 0.00001). According to the funnel plot (Fig. **S4a**), there was no asymmetry; however minor asymmetry existed in the Doi plot (Fig. **S4b**) with an LFK index of -1.82.

#### 3.4.3 FPG

In terms of FPG, a meta-analysis of ten RCTs was conducted. IDegLira can reduce the level of FPG compared with the control group (MD = −0.90; 95% CI [−1.40, −0.^41^]; *p* = 0.0003) (Fig. **S5A**). According to the sensitivity analysis, the results were robust. The subgroup analysis also yielded similar results (Fig. **S5B**). The funnel plot (Fig. **S6a**) did not display any publication bias, whereas the Doi plot displayed minor asymmetry with the LFK index of -0.50 (Fig. **S6b**).

#### 3.4.4 SMPG

SMPG data were reported in eight RCTs. IDegLira significantly reduced SMPG (MD = −0.82; 95% CI [−1.22, −0.^42^]; *p* < 0.0001) (Fig. **S7A**) compared with control groups. Sensitivity analysis showed that the results were robust. The subgroup analysis of IDegLira *versus* different control groups yielded similar results (Fig. **S7B**). For publication bias, there was no asymmetry in the funnel plot (Fig. **S8a**), but the Doi plot (Fig. **S8b**) showed minor asymmetry with the LFK index of -0.65.

#### 3.4.5 Target Glycemic Control and Composite Endpoint of HbA1c

##### 3.4.5.1 Target Glycemic Control

All RCTs reported the proportion of participants achieving the HbA1c level of < 7.0% or < 6.5%. Compared with control groups, IDegLira had a larger proportion of participants achieving the HbA1c level of < 7.0% (RR = 1.66; 95% CI [1.44, 1.92]; *p* < 0.00001) (Fig. **4A**) or < 6.5% (RR = 2.13; 95% CI [1.76, 2.57]; *p* < 0.00001) (Fig. **4B**). Sensitivity analysis indicated that the results were robust. We obtained similar results in the subgroup analysis (Fig. **S9A** and **S9B**). It was noted that the funnel plots (Fig. **S10a**) for meeting the HbA1c target of < 7.0% were slightly asymmetrical, and the Doi plot (Fig. **S10b**) displayed major asymmetry with the LFK index of 5.06. Similarly, the funnel plots (Fig. **S11a**) for achieving the HbA1c target of < 6.5% were slightly asymmetrical, and the Doi plot (Fig. **S11b**) indicated major asymmetry with an LFK index of 4.92. Thus, we conducted a non-parametric trim and fill analysis. After incorporating two additional imputed studies, the adjusted pooled proportion of participants achieving the HbA1c level of < 7.0% was 1.520 (95% CI: 1.329, 1.738) (Fig. **S12A**). However, there were no imputed missing studies in achieving the HbA1c level of < 6.5%, and the overall pooled proportion of participants achieving the HbA1c level of < 6.5% was 2.263 (95% CI: 1.678, 3.053) (Fig. **S12B**).

##### 3.4.5.2 Composite Endpoint of HbA1c

Four RCTs reported the data of achieving the target of HbA1c < 7.0% or < 6.5% without hypoglycemia and weight gain. From the forest plot (Fig. **5A** and **B**), there were no statistically significant differences between IDegLira and control drugs in the composite endpoint. However, it is worth noting that different results were obtained in sensitivity analysis and subgroup analysis. In sensitivity analysis, one study [17] significantly altered the overall effect results. In subgroup analysis, IDegLira were superior to insulin in achieving the target of HbA1c < 7.0 or < 6.5% without hypoglycemia and weight gain (Fig. **5C** and **D**). For publication bias, the funnel plot (Fig. **S13a**) and the Doi plot (Fig. **S13b**) showed no asymmetry in achieving the target of HbA1c < 7.0% without hypoglycemia and weight gain. Likewise, there was no asymmetry in the funnel plot (Fig. **S14a**) and Doi plot (Fig. **S14b**) for achieving the target of HbA1c < 6.5% without hypoglycemia and weight gain.

### 3.5 Meta-Analysis of Safety Outcomes

#### 3.5.1 Hypoglycemia

In all, six studies reported the incidence of hypoglycemia. Compared with control groups, the incidence of hypoglycemia with IDegLira didn't show statistically significant differences (RR = 0.83; 95% CI [0.49, 1.^40^]; *p* = 0.48) (Fig. **S15A**). Sensitivity analysis demonstrated the results were robust. However, it is worth noting that different results were obtained in the subgroup analysis. Compared with insulin, IDegLira decreased the number of participants who experienced hypoglycemia (RR = 0.64; 95% CI [0.46, 0.88]; *p* = 0.007), but increased the number (RR = 15.27; 95% CI [5.63, 41.^42^]; *p* < 0.00001) compared with GLP-1 RA (Fig. **S15B**). For publication bias, a slight asymmetry was observed in the funnel plot (Fig. **S16a**), and the Doi plot showed major asymmetry with an LFK index of 2.02 (Fig. **S16b**). There were no imputed missing studies in the incidence of hypoglycemia, and the pooled incidence of hypoglycemia was 0.697 (95% CI: 0.589, 0.825) (Fig. **S17**).

#### 3.5.2 Adverse Events and Serious Adverse Events

Ten RCTs on AEs and eleven RCTs on SAEs events were conducted meta-analysis. There were no statistically significant differences between IDegLira and Control drugs in the incidence of AEs (RR = 0.99; 95% CI [0.96, 1.0^3^]; *p* = 0.77) (Fig. **S18A**) and SAEs (RR = 0.92; 95% CI [0.72, 1.^19^]; *p* = 0.54) (Fig. **S18B**). Sensitivity analysis showed the results were robust. We obtained similar results in the subgroup analysis of IDegLira *versus* different control groups (Fig. **S19A** and **S19B**). In publication bias, it was observed that the funnel plot (Fig. **S20a**) was slightly asymmetric, whereas the Doi plot (Fig. **S20b**) had a major asymmetry with the LFK index of 2.43 in AEs; For SAEs, there was no asymmetry in the funnel plot (Fig. **S21a**) and the Doi plot (Fig. **S21b**) with the LFK index of 0.58. Considering this significant publication bias in AEs, the non-parametric trim and fill analysis was performed. The analysis revealed that three imputed missing studies were identified in AEs, and the adjusted overall incidence of AEs was 0.984 (95% CI: 0.954, 1.016) (Fig. **S22**).

### 3.6 Meta-regression

We performed meta-regression to explore additional sources of heterogeneity. As shown in Table **2**, there was no significant association between baseline variables, such as age, sex ratio, baseline HbA1c, body weight, and diabetes duration, and the change in HbA1c or achieving the target of HbA1c < 7.0%. Nonetheless, it was worth noting that baseline HbA1c had a significant impact on achieving the target of HbA1c < 6.5%. Additionally, blinding contributed significantly to the heterogeneity of these outcomes.

## 4 DISCUSSION

Most T2DM patients typically featured with overweight and cardiovascular disease (CVD) comorbidities. Apart from lifestyle management, individualized medicinal treatment should be carefully formulated for a good outcome. In the present study, 12 RCTs of IDegLira in treating adult patients with T2DM were synthesized for meta-analysis. According to the overall analysis, sensitivity analysis, and subgroup analysis, IDegLira exhibited advantages in reducing HbA1c, FPG, and SMPG, with nearly equal safety outcomes compared to the control groups. Furthermore, IDegLira treatment achieved the target of HbA1c < 7.0% or < 6.5% without weight gain and hypoglycemia (the composite endpoint). This subgroup analysis on its safety showed that IDegLira reduces some of the key adverse effects, especially those related to body weight gain and the risk of hypoglycemia associated with basal insulin alone. Therefore, IDegLira seems to be a better treatment option for adult patients with T2DM who are overweight and unable to control HbA1c levels effectively by oral antidiabetic drugs (OAD) like metformin or its combination. The drug instruction also recommends IDegLira to those who failed to effectively achieve target HbA1c levels with injection insulin combined with OAD or GLP-1 RA combined with OAD.

An individualized treatment regimen for T2DM is established, considering the complications, especially those related to CVD. Liraglutide, the main component in IDegLira, showed a beneficial effect on coronary heart disease, including coronary syndromes (ACS and CCS), atherosclerotic cardiovascular disease (ASCVD), and Percutaneous Coronary Intervention (PCI). T2DM patients with hypertension can benefit from improvements in systolic blood pressure and lipid profiles (total cholesterol, very low-density lipoprotein cholesterol) [21].

In order to prevent long-term diabetes complications, controlling individualized glycemic target levels is a significant issue. The American Diabetes Association (ADA) recommended a general target HbA1c level of 7.0%, while a more stringent HbA1c level of 6.5% or lower is recommended as long as it can be achieved safely by the American Association of Clinical Endocrinology (AACE) [26, 27]. However, it should be noted that most antihyperglycemic agents could bring potential risks, especially in terms of body weight gain and hypoglycemia [28], which prompts the reconstruction of a composite endpoint for medication consideration in terms of therapeutic efficacy and adverse effects. In this context, the explicit integration of individualized HbA1c goals with body weight and hypoglycemia risks as dual endpoints could serve as a good guideline. Moreover, the composite endpoint should be balance between efficacy and safety based on a stratified approach. For example, rigorous targets (≤6.5%) could be prioritized in patients where weight neutrality and avoidance of hypoglycemia are feasible. In comparison, a less stringent target (7.0%) may apply to elderly patients or those with CVD comorbidities. In another case, when a T2DM patient with advanced β-cell dysfunction requires insulin intensification to achieve target HbA1c levels, IDegLira could be a preferred option over basal-bolus regimens to reduce weight loss and hypoglycemia risks. Evidently, a rational composite endpoint integrating HbA1c goals with body weight and hypoglycemia risks would be applicable in the individualized T2DM treatment program with more details consideration.

There is evidence that IDegLira can simultaneously cover the multiple pathogeneses of T2DM. For one thing, by inhibiting hepatic glucose production, inhibiting lipolysis and increasing glucose consumption, insulin degludec can exert a stable and long-lasting hypoglycemic effect in target organs like skeletal muscle, liver and fat [29]; for another, liraglutide acts on GLP-1 receptors in target organs such as the brain, gastrointestinal tract and pancreas, improving pancreatic β-cell function, delaying gastric emptying, increasing insulin sensitivity and promoting glucose-dependent insulin secretion [30]. From this mechanistic evidence, IDegLira can simultaneously perform exogenous insulin supplementation and endogenous regulation of pancreatic function. Meanwhile, the collaboration and complementarity between the components yield a potent hypoglycemic effect for those with an unstable blood sugar baseline on OAD.

The DUAL clinical trials indicate that IDegLira not only provides additional glycemic control for T2DM that is uncontrolled on GLP-RA or insulin alone, but also reduces some of the key adverse effects of both insulin and GLP-1 RAs [14, 17, 24]. Especially, IDegLira can help individuals lose weight and reduce the risk of hypoglycemia associated with basal insulin alone [31-33]. Compared to treatment with GLP-1 RAs alone, the incidence of gastrointestinal adverse events is lower [21, 23]. In the present study, IDegLira is apparently superior to single-component control drugs in reducing HbA1c, FPG, and SMPG, and in achieving the target HbA1c level, but AEs and SAEs did not increase. Therefore, IDegLira is a safe candidate for use as a hypoglycemic agent.

The management of type 2 diabetes mellitus is a long-term program. A single antihyperglycemic agent may be appropriate for the initial treatment; however, the progressive nature of the disease will eventually lead patients to resort to a multi-drug combination [34]. Consequently, the complicated regimens and resulting financial expense may impact patients' treatment compliance [35]. As a combination medication, its convenience in self-administration (a single daily injection) helps enhance patients' adherence to the therapeutic schedule [36, 37]. In addition, the cost-effectiveness evaluations based on clinical trial data indicate that IDegLira as a cost-effective therapeutic option outperforms several counterpart regimens (basal insulin up-titration, basal-bolus insulin, GLP-1 receptor agonists, and GLP-1 receptor agonists in combination with insulin) in patients in the United States and European countries [38-43].

## 4 STUDY LIMITATIONS

The present meta-analysis was registered in PROSPERO before the literature search was completed. We followed this pre-published protocol, which ensures transparency to avoid bias. However, several limitations in this meta-analysis must be noted. First, the analysis was based on study-level data rather than Individual Patient Data (IPD), which restricted our ability to perform more granular analyses, such as evaluating treatment effects across different patient populations or exploring differences across subgroups. Second, as with most meta-analysis articles, our systematic review and meta-analysis demonstrated a high level of statistical heterogeneity. Several measures, including subgroup analyses and meta-regression, were conducted to investigate the sources of heterogeneity among studies. The results suggest that blinding of the included RCTs may be the primary factor contributing to this heterogeneity. Double-blind trials are crucial in the context of a meta-analysis, whereas open-label RCTs often introduce performance bias and detection bias. Additionally, trial sponsorship could introduce bias in the data interpretation framework, drawing out sponsor-flavored efficacy indicators. For example, the funding agreement may have predefined efficacy endpoints such as short-term efficacy rather than long-term safety. Generally, sponsors are more likely to publish positive results while ignoring negative or neutral findings. Third, while assessed, publication bias was detected for certain outcomes (*e.g.*, target glycemic control, hypoglycemia, and AEs). Therefore, we conducted a non-parametric trim and fill analysis. The adjusted effect estimates and the actual effect size were largely similar, which suggested that publication bias had no significant impact on the final results. Finally, there was a gap that most studies have a follow-up time of only 26 weeks, which limits insights into the long-term efficacy and safety profile of IDegLira. Hence, it is recommended that further research be conducted with an extended follow-up time in the future. Given these limitations, more homogeneous high-quality RCTs will be needed to validate the results of this systematic review and meta-analysis in the future.

## CONCLUSION

This systematic review and meta-analysis demonstrated that IDegLira has a favorable effect on reducing HbA1c, FPG, and SMPG, while also achieving target glycemic control. Additionally, it helps individuals lose weight and reduce the risk of hypoglycemia associated with basal insulin alone. In summary, IDegLira offers a valuable therapeutic option to patients with T2DM who have not achieved adequate glycemic control with OAD, patients who are overweight and sensitive to hypoglycemia risk. In addition, IDegLira is suitable for patients who have comorbid CVD and those who have an urgent need for effective glycemic management. However, due to some limitations, the current results should be cautiously interpreted for reference.

## Figures and Tables

**Fig. (1):**
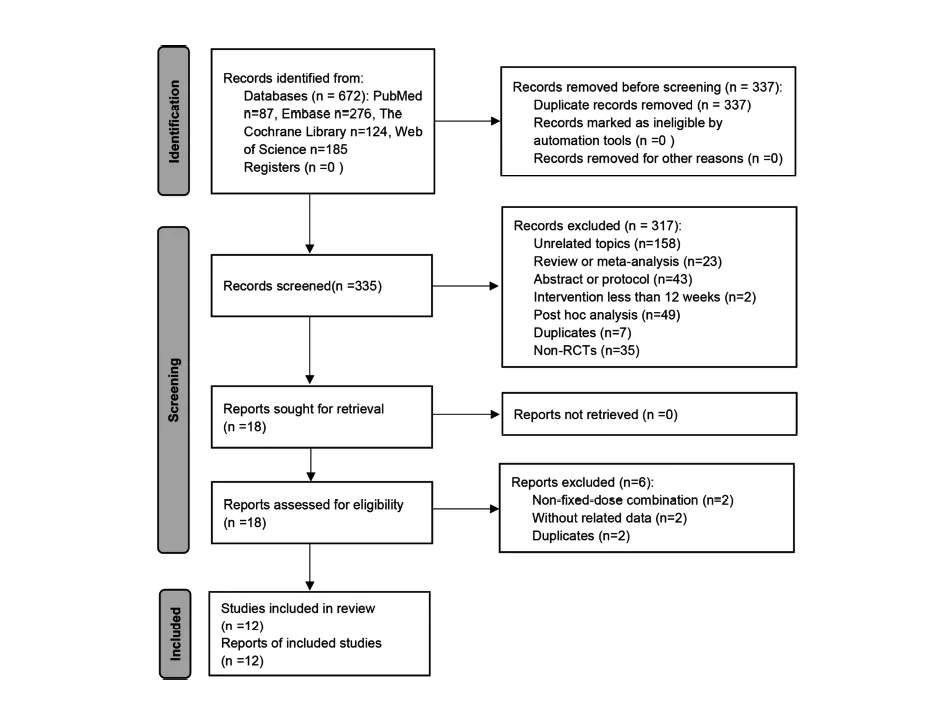
Flow diagram of study selection.

**Fig. (2):**
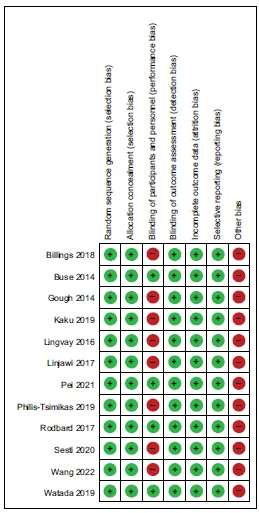
Risk of bias summary: the judgments of the reviewers about each risk of bias item for each included study. Green or + represents that the study is at lower risk of bias. Red or + represents that the study is at high risk of bias.

**Fig. (3):**
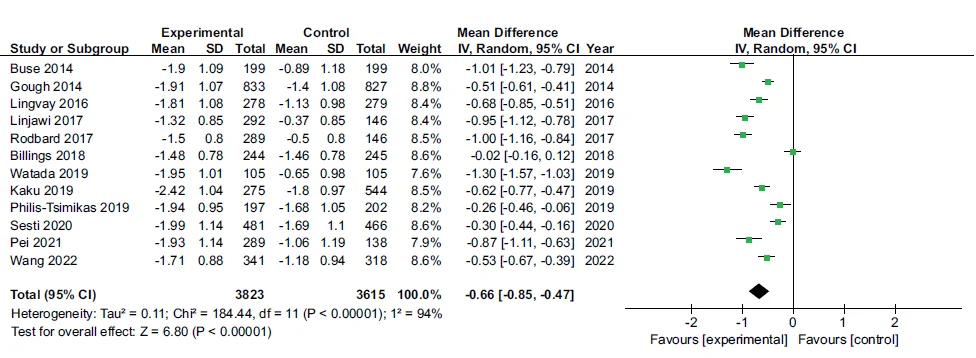
Forest plot of studies evaluating the effect of IDegLira on HbA1c (%). Effect sizes: Mean Difference (MD), heterogeneity metrics: I^2^, error bars represent 95% Confidence Intervals (CIs).

**Fig. (4):**
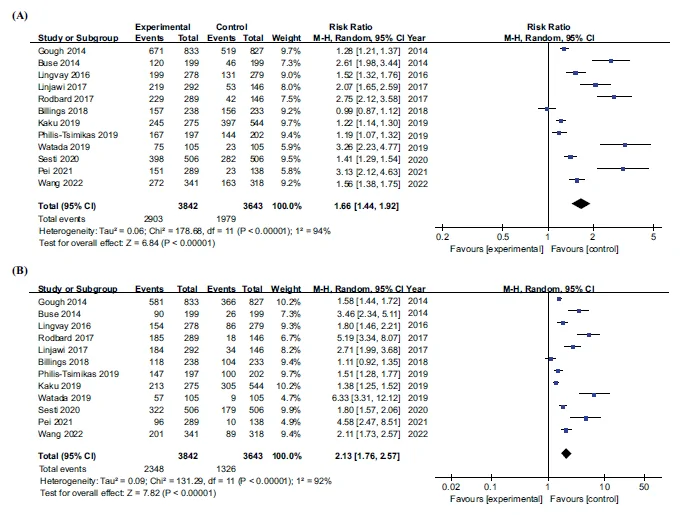
Forest plot of studies evaluating the effect of IDegLira in the target glycemic control: Achieving the target of HbA1c < 7.0% (**A**); Achieving the target of HbA1c < 6.5% (**B**). Effect sizes: risk ratio (RR), heterogeneity metrics: I^2^, error bars represent 95% confidence intervals (CIs).

**Fig. (5):**
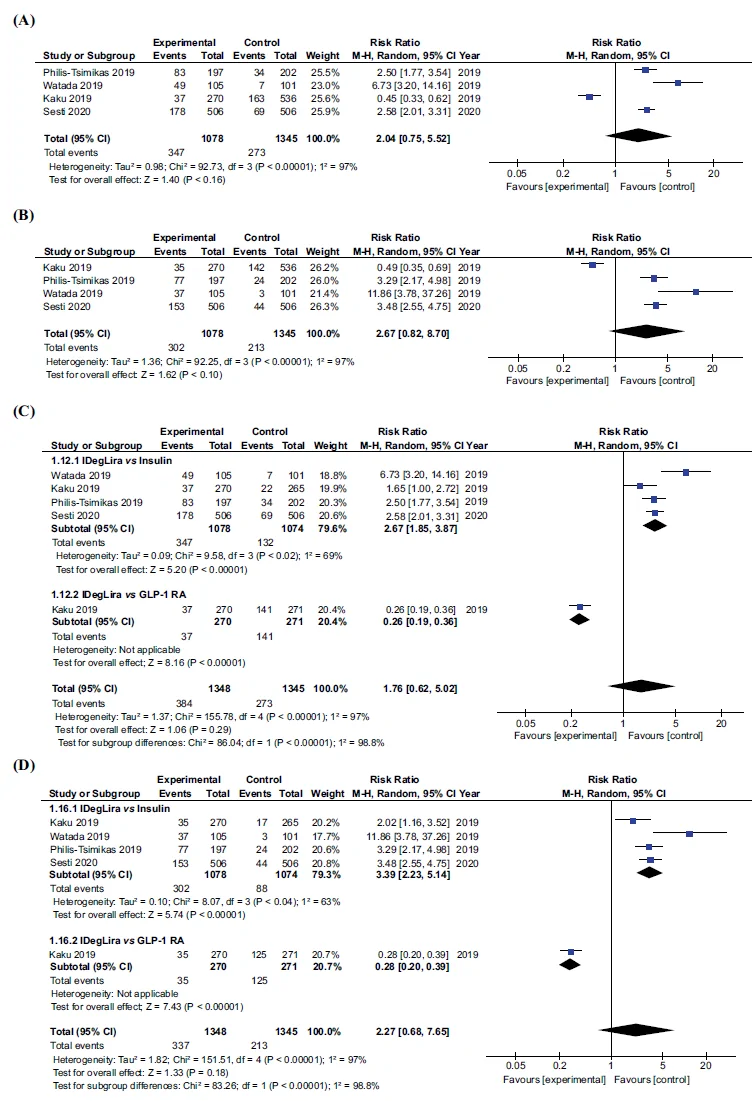
Forest plot of studies evaluating the effect of IDegLira in the composite endpoint of HbA1c: Achieving the target of HbA1c < 7.0% (**A**) or < 6.5% (**B**) without weight gain and hypoglycemia; Forest plot of the subgroup analysis by control types for composite endpoint of HbA1c: Achieving the target of HbA1c < 7.0% (**C**) or < 6.5% (**D**) without weight gain and hypoglycemia (severe or blood glucose-confirmed symptomatic hypoglycemia during a participant's last 12 weeks of treatment).Effect sizes: Risk Ratio (RR), heterogeneity metrics: I^2^, error bars represent 95% Confidence Intervals (CIs).

**Table 1:** Characteristics of the included randomized controlled trials.

**Study: ClinicalTrials.gov Registration no.**	**Country**	**Blinding**	**Background Therapy**	**Study Duration (Weeks)**	**Study Arms**	**n**	**HbA1c (%)**	**Body Weight (kg)**	**Age (Years)**	**Sex, Men, n (%)**	**Diabetes Duration (years)**
Wang *et al, *2022 (DUAL-I China) [14]; NCT03172494	China (multicenter)	Open-label	Met ± other OADs	26	IDegLira Degludec Liraglutide	361 179 180	8.2 ± 0.8 8.3 ± 0.8 8.2 ± 0.8	74.8 ± 14.3 73.2 ± 13.2 73.8 ± 13.3	54.5 ± 10.3 55.7 ± 10.2 54.1 ± 10.2	219(60.7) 100(55.9) 108(60.0)	8.0 ± 5.3 8.6 ± 5.6 8.1 ± 5.3
Pei *et al, *2021 (DUAL-II China) [15]; NCT03175120	China (multicentre)	Double-blind	Insulin + Met ± other OADs	26	IDegLira Degludec	302 151	8.9 ± 1.2 9.0 ± 1.2	76.8 ± 13.0 74.3 ± 11.4	54.5 ± 9.8 55.3 ± 10.0	183(60.6) 91(60.3)	11.5 ± 5.9 11.3 ± 6.3
Sesti *et al, *2020 (DUAL-VIII) [16]; NCT02501161	Multinational	Open-label	Other OADs ± Met	26	IDegLira Glargine	506 506	8.4 ± 1.0 8.6 ± 1.0	89.7 ± 20.5 89.0 ± 20.1	56.8 ± 10.0 56.4 ± 10.1	280(55) 275(54)	10.0 ± 6.2 10.2 ± 6.1
Kaku *et al, *2019 (DUAL- I Japan) [17]; NCT02607306	Japan (multicentre)	Open-label	Other OADs ± Met	52	IDegLira Degludec Liraglutide	275 271 273	8.5 ± 1.1 8.5 ± 1.1 8.3 ± 1.0	70.7 ± 12.4 72.6 ± 14.5 72.2 ± 15.0	56.9 ± 10.2 57.8 ± 9.9 56.8 ± 10.1	194(70.5) 195(72.0) 192(70.3)	9.2 ± 6.2 9.7 ± 6.0 9.4 ± 5.9
Watada *et al, *2019 (DUAL- II Japan) [18]; NCT02911948	Japan (multicentre)	Double-blind	Insulin + Met ± other OADs	26	IDegLira Degludec	105 105	8.6 ± 0.9 8.6 ± 0.8	73.9 ± 11.9 75.5 ± 14.0	56.6 ± 10.4 55.5 ± 10.0	70(66.7) 63(60.0)	14.3 ± 7.8 13.8 ± 7.5
Philis-Tsimikas *et al, *2019(DUAL-IX) [19]; NCT02773368	Multinational	Open-label	SGLT2i ± Met ± other OADs	26	IDegLira Glargine	210 210	8.2 ± 0.9 8.4 ± 1.1	89.3 ± 17.6 87.2 ± 17.2	56.1 ± 10.4 57.2 ± 10.2	121(57.6) 126(60.0)	9.8 ± 6.2 9.3 ± 6.3
Billings *et al, *2018(DUAL-VII) [20]; NCT02420262	Multinational	Open-label	Insulin + Met	26	IDegLira Glargine + IAsp	252 254	8.2 ± 0.8 8.2 ± 0.8	87.2 ± 16.0 88.2 ± 17.2	58.6 ± 9.0 58.0 ± 8.6	110(43.7) 117(46.1)	13.2 ± 7.0 13.3 ± 6.8
Linjawi *et al, *2017(DUAL-III) [21]; NCT01676116	Multinational	Open-label	Met ± other OADs	26	IDegLira Liraglutide or Exenatide	292 146	7.8 ± 0.6 7.7 ± 0.6	95.6 ± 16.6 95.5 ± 17.3	58.3 ± 9.9 58.4 ± 8.8	153(52.4) 71(48.6)	10.4 ± 5.8 10.4 ± 5.8
Rodbard *et al, *2017(DUAL-IV) [22]; NCT01618162	Multinational	Double-blind	SU ± Met	26	IDegLira Placebo	289 146	7.9 ± 0.6 7.9 ± 0.6	87.2 ± 18.6 89.3 ± 17.5	60.0 ± 9.6 59.4 ± 10.8	154(53.3) 73(50.0)	9.0 ± 5.5 9.3 ± 6.5
Lingvay *et al, *2016(DUAL-V) [23]; NCT01952145	Multinational	Open-label	Insulin + Met	26	IDegLira Glargine	278 279	8.4 ± 0.9 8.2 ± 0.9	88.3 ± 17.5 87.3 ± 15.8	58.4 ± 9.8 59.1 ± 9.3	143(51.4) 137(49.1)	11.6 ± 7.4 11.3 ± 6.6
Gough *et al, *2014(DUAL-I) [24]; NCT01336023	Multinational	Open-label	Met ± PIO	26	IDegLira Degludec Liraglutide	833 413 414	8.3 ± 0.9 8.3 ± 1.0 8.3 ± 0.9	87.2 ± 19.0 87.4 ± 19.2 87.4 ± 18.0	55.1 ± 9.9 54.9 ± 9.7 55.0 ± 10.2	435(52.2) 200(48.4) 208(50.2)	6.6 ± 5.1 7.0 ± 5.3 7.2 ± 6.1
Buse *et al, *2014 (DUAL-II) [25]; NCT01392573	Multinational	Double-blind	Insulin + Met ± other OADs	26	IDegLira Degludec	199 199	8.7 ± 0.7 8.8 ± 0.7	95.4 ± 19 93.5 ± 20	57.0 ± 9.0 58.0 ± 11.0	111(56) 105(53)	10.0 ± 6.0 11.0 ± 7.0

**Abbreviations: **NCT: National Clinical Trial number; n: number of participants; HbA1c: hemoglobin A1c; Met: metformin; OAD: oral antidiabetic drug; SGLT2i: sodium-glucose cotransporter 2 inhibitors; SU: sulphonylurea; PIO: pioglitazone; IAsp: insulin aspart.

**Table 2:** Meta-regression analysis of factors affecting changes in HbA1c and target glycemic control.

**Outcome**	**Independent Variable**	**Coefficient, 95%CI**	***p*-value**
HbA1c	Age, years	0.04 (-0.21,0.29)	0.693
	Sex, men, n (%)	-0.03 (-0.09,0.03)	0.248
	Baseline HbA1c (%)	0.93 (-0.30,2.16)	0.108
	Body weight (kg)	-0.01 (-0.07,0.04)	0.541
	Diabetes duration (years)	-0.02 (-0.18,0.14)	0.735
	Blinding	0.75 (0.17,1.32)	0.020
Target glycemic control (achieving the target of HbA1c < 7.0%) Target glycemic control (achieving the target of HbA1c < 6.5%)	Age, years Sex, men, n (%) Baseline HbA1c (%) Body weight (kg) Diabetes duration (years) Blinding Age, years Sex, men, n (%) Baseline HbA1c (%) Body weight (kg) Diabetes duration (years) Blinding	-0.17 (-0.46,0.13) 0.02 (-0.04,0.09) -0.94. (-2.40,0.52) 0.02 (-0.04,0.08) 0.09 (-0.09,0.28) -1.67 (-2.36,-0.99) -0.37 (-0.88,0.14) 0.04 (-0.07,0.16) -2.91 (-5.42,-0.41) 0.02 (-0.09,0.13) 0.24 (-0.08,0.56) -3.61 (-4.80,-2.43)	0.203 0.397 0.160 0.437 0.253 0.001 0.123 0.341 0.031 0.661 0.114 0.001

**Abbreviations: **CI: confidence intervals. Blinding: double-blind or open-label.

## Data Availability

Not applicable.

## LIST OF ABBREVIATIONS

T2DM: Type 2 Diabetes Mellitus
RR: Risk Ratio
CI: Confidence Intervals
MD: Mean Difference
IDF: International Diabetes Federation
IR: Insulin Resistance
HbA1c: Hemoglobin A1C
GLP-1 RAs: Glucagon-like Peptide-1 Receptor Agonists
FPG: Fasting Plasma Glucose
RCTS: Randomized Controlled Trials
PRISMA: Preferred Reporting Items for Systematic Reviews and Meta-Analyses
PICOS: Participants, Interventions, Comparisons, Outcomes, and Study Design
MESH: Medical Subject Headings
SMPG: Self-Measured Plasma Glucose
AES: Adverse Events
NCT: National Clinical Trial
CVD: Cardiovascular Disease
OAD: Oral Antidiabetic Drugs
ASCVD: Atherosclerotic Cardiovascular Disease
PCI: Percutaneous Coronary Intervention
ADA: American Diabetes Association
AACE: American Association of Clinical Endocrinology
IPD: Individual Patient Data

## STANDARDS OF REPORTING

PRISMA guidelines and methodology were followed.

## SUPPLEMENTARY MATERIAL

PRISMA checklist is available as supplementary material on the publisher’s website along with the published article. Supplementary material is available on the publisher’s website along with the published article.
