# Supplementary material for: Efficacy and Safety of IDegLira in Patients with Type 2 Diabetes Mellitus: A Systematic Review and Meta-analysis of Randomized Controlled Trials
Source: Curr Diabetes Rev. 2025 Sep 11;22(5):E15733998359708. doi: 10.2174/0115733998359708250822102217 (PMC13714749; doi:10.2174/0115733998359708250822102217)
Supplement: Supplementary file 1 [file CDR-22-5-E15733998359708_SD1.pdf]

Supplementary Material

Efficacy and Safety of IDegLira in Patients with Type 2 Diabetes Mellitus: A Systematic Review and Meta-analysis of Randomized Controlled Trials

Lexie Bai[1], Delong Liu[1], Ning Su[1], Lina Zhang[1] and Zhiyong Yang[1,*]

[1]*Pharmacy College, Dali University, Dali, 671000, China*

1. LITERATURE SEARCH

1.1. Pubmed Search Strategy

#1 Diabetes Mellitus, Type 2[MeSH Terms]

#2 Diabetes Mellitus, Noninsulin-Dependent[Title/Abstract] OR Diabetes Mellitus, Ketosis-Resistant[Title/Abstract] OR Diabetes Mellitus, Ketosis Resistant[Title/Abstract] OR Ketosis-Resistant Diabetes Mellitus[Title/Abstract] OR Diabetes Mellitus, Non Insulin Dependent[Title/Abstract] OR Diabetes Mellitus, Non-Insulin-Dependent[Title/Abstract] OR Non-Insulin-Dependent Diabetes Mellitus[Title/Abstract] OR Diabetes Mellitus, Stable[Title/Abstract] OR Stable Diabetes Mellitus[Title/Abstract] OR Diabetes Mellitus, Type II[Title/Abstract] OR NIDDM[Title/Abstract] OR Diabetes Mellitus, Noninsulin Dependent[Title/Abstract] OR Diabetes Mellitus, Maturity-Onset[Title/Abstract] OR Diabetes Mellitus, Maturity Onset[Title/Abstract] OR Maturity-Onset Diabetes Mellitus[Title/Abstract] OR Maturity Onset Diabetes Mellitus[Title/Abstract] OR MODY[Title/Abstract] OR Diabetes Mellitus, Slow-Onset[Title/Abstract] OR Diabetes Mellitus, Slow Onset[Title/Abstract] OR Slow-Onset Diabetes Mellitus[Title/Abstract] OR Type 2 Diabetes Mellitus[Title/Abstract] OR Noninsulin-Dependent Diabetes Mellitus[Title/Abstract] OR Noninsulin Dependent Diabetes Mellitus[Title/Abstract] OR Maturity-Onset Diabetes[Title/Abstract] OR Diabetes, Maturity-Onset[Title/Abstract] OR Maturity Onset Diabetes[Title/Abstract] OR Type 2 Diabetes[Title/Abstract] OR Diabetes, Type 2[Title/Abstract] OR Diabetes Mellitus, Adult-Onset[Title/Abstract] OR Adult-Onset Diabetes Mellitus[Title/Abstract] OR Diabetes Mellitus, Adult Onset[Title/Abstract]

#3 #1OR#2

#4 IDegLira[Supplementary Concept]

#5 insulin degludec/liraglutide OR Xultophy [Title/Abstract]

#6 #4OR#5

#7 Randomized Controlled Trials as Topic[MeSH Terms]

#8 Controlled Clinical Trials as Topic[MeSH Terms]

#9 Clinical Trials, Randomized[Title/Abstract] OR Trials, Randomized Clinical[Title/Abstract] OR Controlled Clinical Trials, Randomized[Title/Abstract] OR randomized controlled trial[Title/Abstract] OR randomized controlled trials[Title/Abstract] OR RCT[Title/Abstract] OR controlled clinical trial[Title/Abstract] OR controlled clinical trials[Title/Abstract] OR random*[Title/Abstract] OR control*[Title/Abstract] OR blind[Title/Abstract] OR blind*[Title/Abstract] OR placebo[Title/Abstract]

#10 #7OR#8OR#9

#11 #3AND#6AND#10

2. EMBASE SEARCH STRATEGY

#1 'diabetes mellitus, type 2'/exp

#2 'diabetes mellitus, noninsulin-dependent':ab,ti OR 'diabetes mellitus, ketosis-resistant':ab,ti OR 'diabetes mellitus, ketosis resistant':ab,ti OR 'ketosis-resistant diabetes mellitus':ab,ti OR 'diabetes mellitus, non insulin dependent':ab,ti OR 'diabetes mellitus, non-insulin-dependent':ab,ti OR 'non-insulin-dependent diabetes mellitus':ab,ti OR 'diabetes mellitus, stable':ab,ti OR 'stable diabetes mellitus':ab,ti OR 'diabetes mellitus, type ii':ab,ti OR niddm:ab,ti OR 'diabetes mellitus, noninsulin dependent':ab,ti OR 'diabetes mellitus, maturity-onset':ab,ti OR 'diabetes mellitus, maturity onset':ab,ti OR 'maturity-onset diabetes mellitus':ab,ti OR 'maturity onset diabetes mellitus':ab,ti OR mody:ab,ti OR 'diabetes mellitus, slow-onset':ab,ti OR 'diabetes mellitus, slow onset':ab,ti OR 'slow-onset diabetes mellitus':ab,ti OR 'type 2 diabetes mellitus':ab,ti OR 'noninsulin-dependent diabetes mellitus':ab,ti OR 'noninsulin dependent diabetes mellitus':ab,ti OR 'maturity-onset diabetes':ab,ti OR 'diabetes, maturity-onset':ab,ti OR 'maturity onset diabetes':ab,ti OR 'type 2 diabetes':ab,ti OR 'diabetes, type 2':ab,ti OR 'diabetes mellitus, adult-onset':ab,ti OR 'adult-onset diabetes mellitus':ab,ti OR 'diabetes mellitus, adult onset':ab,ti

#3 #1OR#2

#4 'ideglira'/exp

#5 'insulin degludec/liraglutide':ab,ti OR **'xultophy':ab,ti**

#6 #4OR #5

#7 'randomized controlled trials as topic'/exp

#8 'controlled clinical trials as topic'/exp

#9 'randomized controlled trials as topic':ab,ti OR 'controlled clinical trials as topic':ab,ti OR 'clinical trials, randomized':ab,ti OR 'trials, randomized clinical':ab,ti OR 'controlled clinical trials, randomized':ab,ti OR 'randomized controlled trial':ab,ti OR 'randomized controlled trials':ab,ti OR rct:ab,ti OR 'controlled clinical trial':ab,ti OR 'controlled clinical trials':ab,ti OR random*:ab,ti OR control*:ab,ti OR blind:ab,ti OR blind*:ab,ti OR placebo:ab,ti

#10 #7OR#8OR#9

#11 #3AND#6AND#10

3. COCHRANE SEARCH STRATEGY

#1 MeSH descriptor: [Diabetes Mellitus, Type 2] explode all trees

#2 (Diabetes Mellitus, Noninsulin-Dependent OR Diabetes Mellitus, Ketosis-Resistant OR Diabetes Mellitus, Ketosis Resistant OR Ketosis-Resistant Diabetes Mellitus OR Diabetes Mellitus, Non Insulin Dependent OR Diabetes Mellitus, Non-Insulin-Dependent OR Non-Insulin-Dependent Diabetes Mellitus OR Diabetes Mellitus, Stable OR Stable Diabetes Mellitus OR Diabetes Mellitus, Type II OR NIDDM OR Diabetes Mellitus, Noninsulin Dependent OR Diabetes Mellitus, Maturity-Onset OR Diabetes Mellitus, Maturity Onset OR Maturity-Onset Diabetes Mellitus OR Maturity Onset Diabetes Mellitus OR MODY OR Diabetes Mellitus, Slow-Onset OR Diabetes Mellitus, Slow Onset OR Slow-Onset Diabetes Mellitus OR Type 2 Diabetes Mellitus OR Noninsulin-Dependent Diabetes Mellitus OR Noninsulin Dependent Diabetes Mellitus OR Maturity-Onset Diabetes OR Diabetes, Maturity-Onset OR Maturity Onset Diabetes OR Type 2 Diabetes OR Diabetes, Type 2 OR Diabetes Mellitus, Adult-Onset OR Adult-Onset Diabetes Mellitus OR Diabetes Mellitus, Adult Onset):ti,ab,kw

#3 #1OR#2

#4 MeSH descriptor: [IDegLira as Topic] explode all trees

#5 (insulin degludec/liraglutide OR Xultophy):ti,ab,kw

#6 #4OR#5

#7 MeSH descriptor: [Randomized Controlled Trials as Topic] explode all trees

#8 MeSH descriptor: [Controlled Clinical Trials as Topic] explode all trees

#9 (Clinical Trials, Randomized OR Trials, Randomized Clinical OR Controlled Clinical Trials, Randomized OR randomized controlled trial OR randomized controlled trials OR RCT OR controlled clinical trial OR controlled clinical trials OR random* OR control* OR blind OR blind* OR placebo):ti,ab,kw

#10 #7OR#8OR#9

#11 #3AND#6AND#10

4. WEB OF SCIENCE SEARCH STRATEGY

#1 TS=(Diabetes Mellitus, Type 2 OR Diabetes Mellitus, Noninsulin-Dependent OR Diabetes Mellitus, Ketosis-Resistant OR Diabetes Mellitus, Ketosis Resistant OR Ketosis-Resistant Diabetes Mellitus OR Diabetes Mellitus, Non Insulin Dependent OR Diabetes Mellitus, Non-Insulin-Dependent OR Non-Insulin-Dependent Diabetes Mellitus OR Diabetes Mellitus, Stable OR Stable Diabetes Mellitus OR Diabetes Mellitus, Type II OR NIDDM OR Diabetes Mellitus, Noninsulin Dependent OR Diabetes Mellitus, Maturity-Onset OR Diabetes Mellitus, Maturity Onset OR Maturity-Onset Diabetes Mellitus OR Maturity Onset Diabetes Mellitus OR MODY OR Diabetes Mellitus, Slow-Onset OR Diabetes Mellitus, Slow Onset OR Slow-Onset Diabetes Mellitus OR Type 2 Diabetes Mellitus OR Noninsulin-Dependent Diabetes Mellitus OR Noninsulin Dependent Diabetes Mellitus OR Maturity-Onset Diabetes OR Diabetes, Maturity-Onset OR Maturity Onset Diabetes OR Type 2 Diabetes OR Diabetes, Type 2 OR Diabetes Mellitus, Adult-Onset OR Adult-Onset Diabetes Mellitus OR Diabetes Mellitus, Adult Onset)

#2 TS=(ideglira OR insulin degludec/liraglutide OR **xultophy**)

#3 TS=(Randomized Controlled Trials as Topic OR Controlled Clinical Trials as Topic OR Clinical Trials, Randomized OR Trials, Randomized Clinical OR Controlled Clinical Trials, Randomized OR randomized controlled trial OR randomized controlled trials OR RCT OR controlled clinical trial OR controlled clinical trials OR random* OR control* OR blind OR blind* OR placebo)

#4 #1AND#2AND#3

Study or Subgroup	Experimental Mean	SD	Total	Control Mean	SD	Total	Weight	Mean Difference IV, Random, 95% CI	Year
**1.2.1 IDegLira vs Insulin**									
Gough 2014	-1.91	1.07	833	-1.44	1.03	413	7.0%	-0.47 [-0.59, -0.35]	2014
Buse 2014	-1.9	1.09	199	-0.89	1.18	199	6.4%	-1.01 [-1.23, -0.79]	2014
Lingvay 2016	-1.81	1.08	278	-1.13	0.98	279	6.7%	-0.68 [-0.85, -0.51]	2016
Billings 2018	-1.48	0.78	244	-1.46	0.78	245	6.9%	-0.02 [-0.16, 0.12]	2018
Philis-Tsimikas 2019	-1.94	0.95	197	-1.68	1.05	202	6.6%	-0.26 [-0.46, -0.06]	2019
Watada 2019	-1.95	1.01	105	-0.65	0.98	105	6.0%	-1.30 [-1.57, -1.03]	2019
Kaku 2019	-2.42	1.04	275	-1.8	1.02	271	6.7%	-0.62 [-0.79, -0.45]	2019
Sesti 2020	-1.99	1.14	481	-1.69	1.1	466	6.9%	-0.30 [-0.44, -0.16]	2020
Pei 2021	-1.93	1.14	289	-1.06	1.19	138	6.3%	-0.87 [-1.11, -0.63]	2021
Wang 2022	-1.71	0.88	341	-1.2	0.99	167	6.7%	-0.51 [-0.69, -0.33]	2022
**Subtotal (95% CI)**			**3242**			**2485**	**66.1%**	**-0.59 [-0.80, -0.38]**	
Heterogeneity: Tau² = 0.10; Chi² = 128.92, df = 9 (P < 0.00001); I² = 93%									
Test for overall effect: Z = 5.59 (P < 0.00001)									
**1.2.2 IDegLira vs GLP-1 RA**									
Gough 2014	-1.91	1.07	833	-1.28	1.13	414	6.9%	-0.63 [-0.76, -0.50]	2014
Linjawi 2017	-1.32	0.85	292	-0.37	0.85	146	6.7%	-0.95 [-1.12, -0.78]	2017
Kaku 2019	-2.42	1.04	275	-1.8	0.92	273	6.8%	-0.62 [-0.78, -0.46]	2019
Wang 2022	-1.71	0.88	341	-1.16	0.89	151	6.7%	-0.55 [-0.72, -0.38]	2022
**Subtotal (95% CI)**			**1741**			**984**	**27.2%**	**-0.69 [-0.85, -0.52]**	
Heterogeneity: Tau² = 0.02; Chi² = 13.14, df = 3 (P = 0.004); I² = 77%									
Test for overall effect: Z = 8.16 (P < 0.00001)									
**1.2.3 IDegLira vs placebo**									
Rodbard 2017	-1.5	0.8	289	-0.5	0.8	146	6.8%	-1.00 [-1.16, -0.84]	2017
**Subtotal (95% CI)**			**289**			**146**	**6.8%**	**-1.00 [-1.16, -0.84]**	
Heterogeneity: Not applicable									
Test for overall effect: Z = 12.31 (P < 0.00001)									
**Total (95% CI)**			**5272**			**3615**	**100.0%**	**-0.64 [-0.80, -0.49]**	
Heterogeneity: Tau² = 0.09; Chi² = 186.72, df = 14 (P < 0.00001); I² = 93%									
Test for overall effect: Z = 8.01 (P < 0.00001)									
Test for subgroup differences: Chi² = 11.70, df = 2 (P = 0.003), I² = 82.9%									

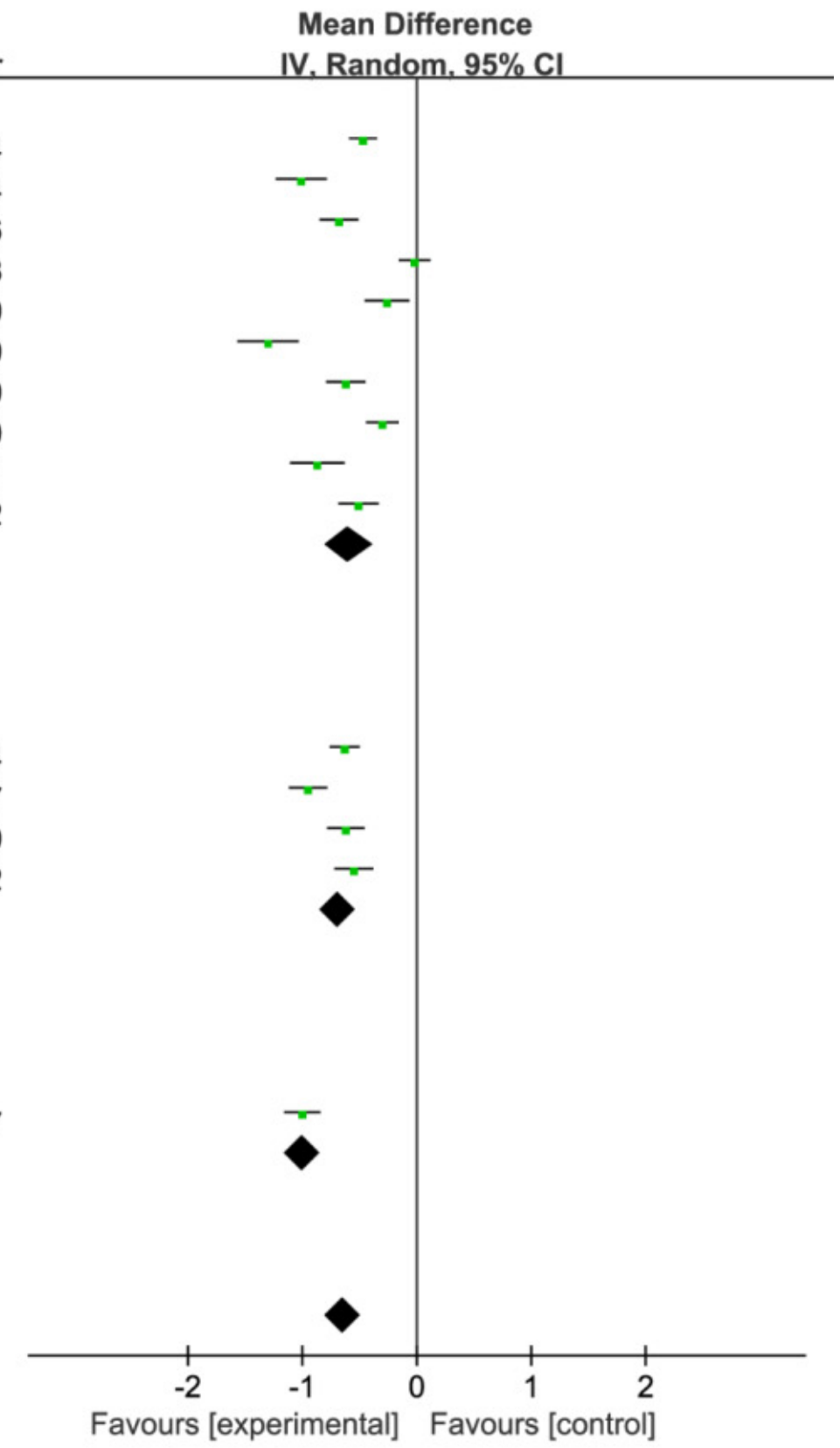

Fig. (S1). Forest plot of the subgroup analysis by control types for evaluating the effect of IDegLira in HbA1c (%). Effect sizes: mean difference (MD), heterogeneity metrics: I², error bars represent 95% confidence intervals (CIs).

(a)

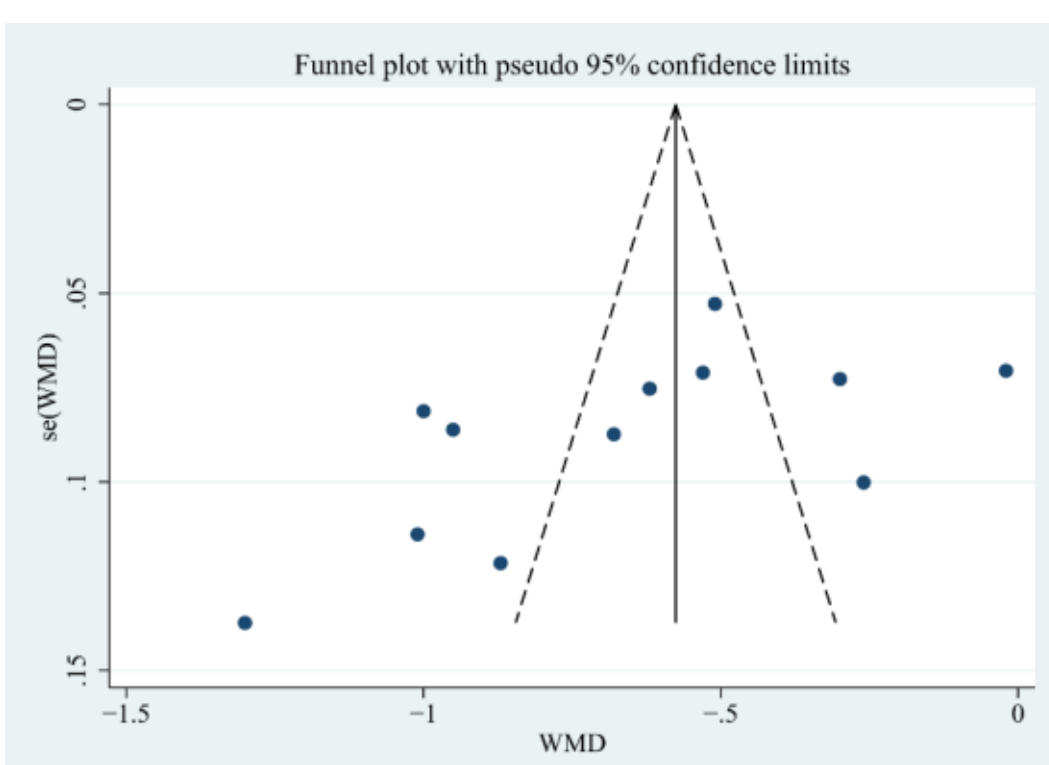

(b)

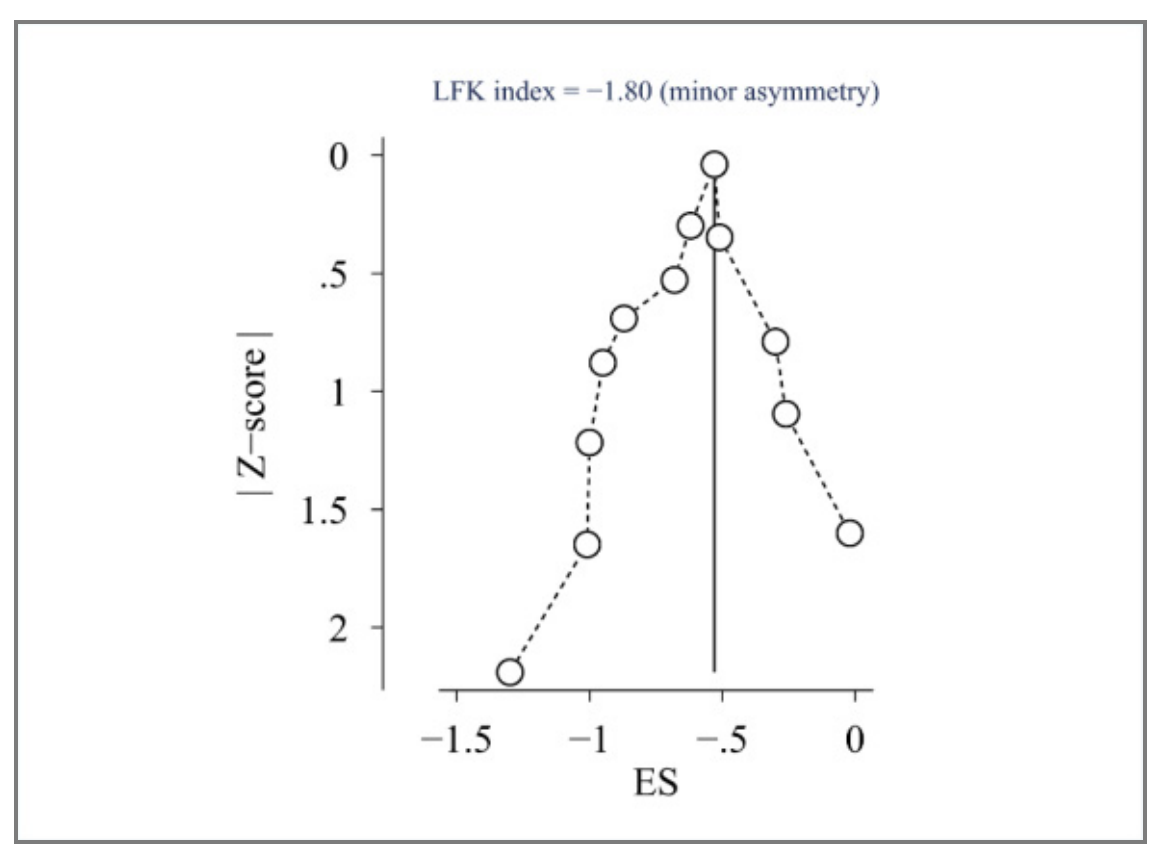

Fig. (S2). Funnel plot and Doi plot of studies evaluating the effect of IDegLira on HbA1c: (**a**) Funnel plot. (**b**) Doi plot. WMD: weighted mean difference, se: standard error, ES: effect size.

(A)

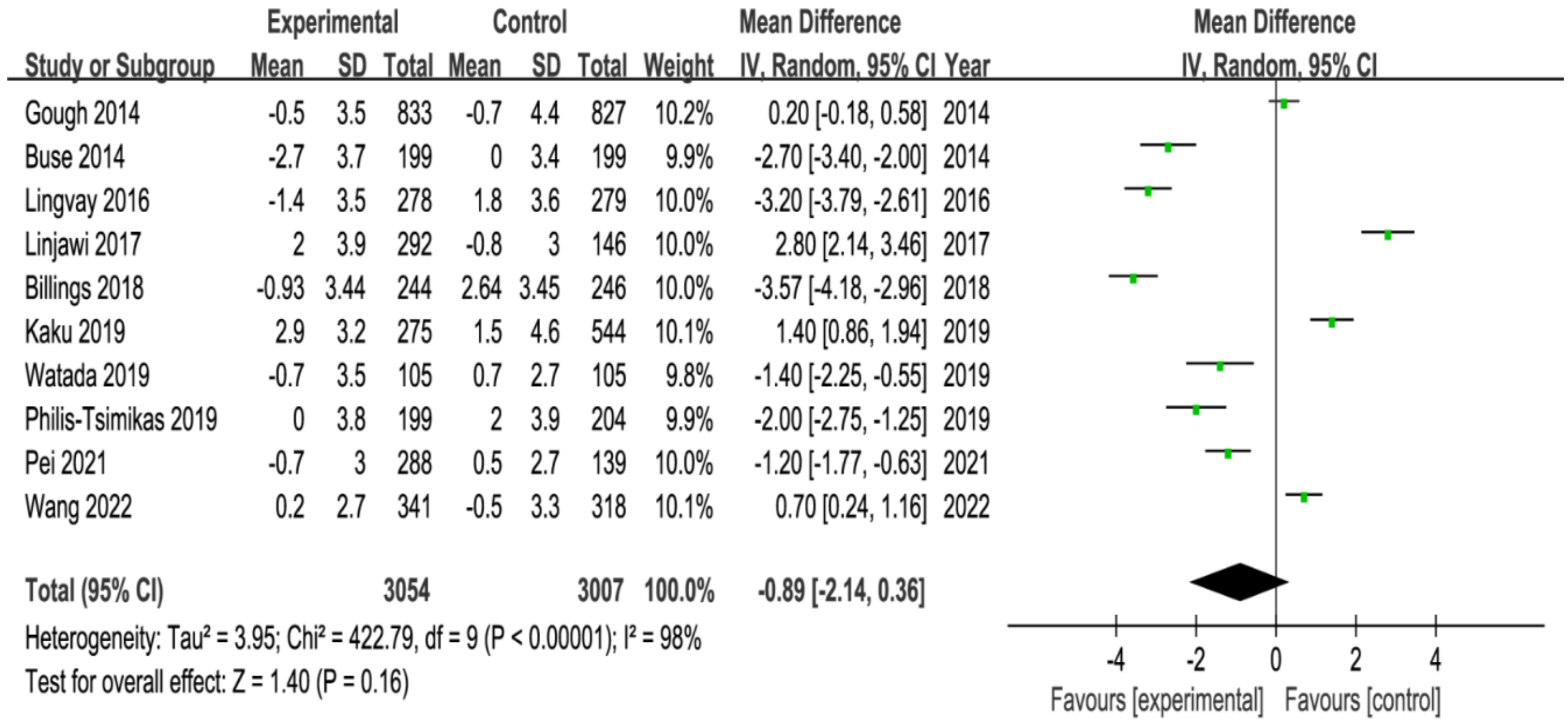

(B)

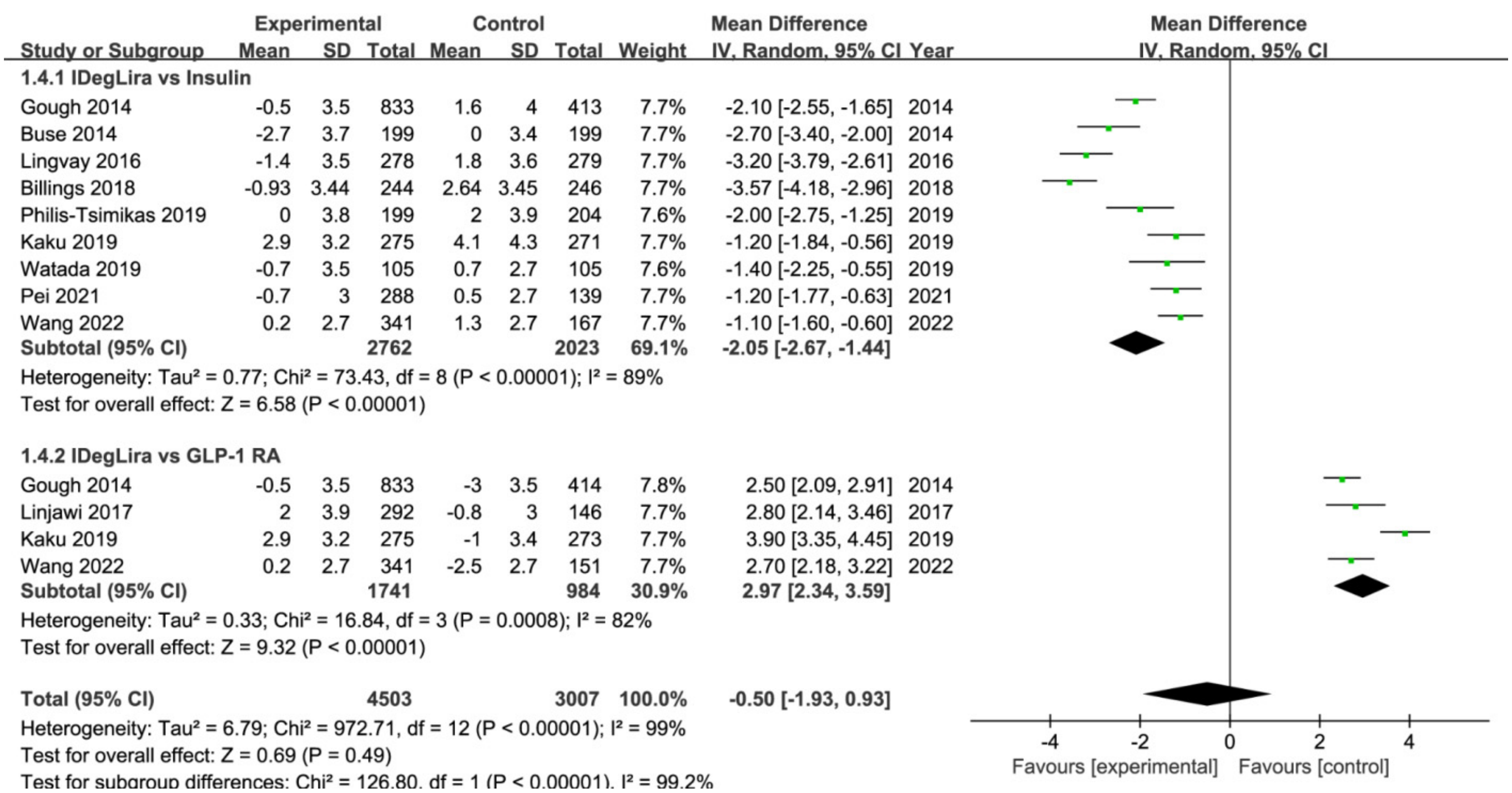

Fig. (S3). (**A**) Forest plot of studies evaluating the effect of IDegLira in body weight (kg). (**B**) Forest plot of the subgroup analysis by control types for evaluating the effect of IDegLira in body weight (kg). Effect sizes: mean difference (MD), heterogeneity metrics: I², error bars represent 95% confidence intervals (CIs).

(a) **(b)**

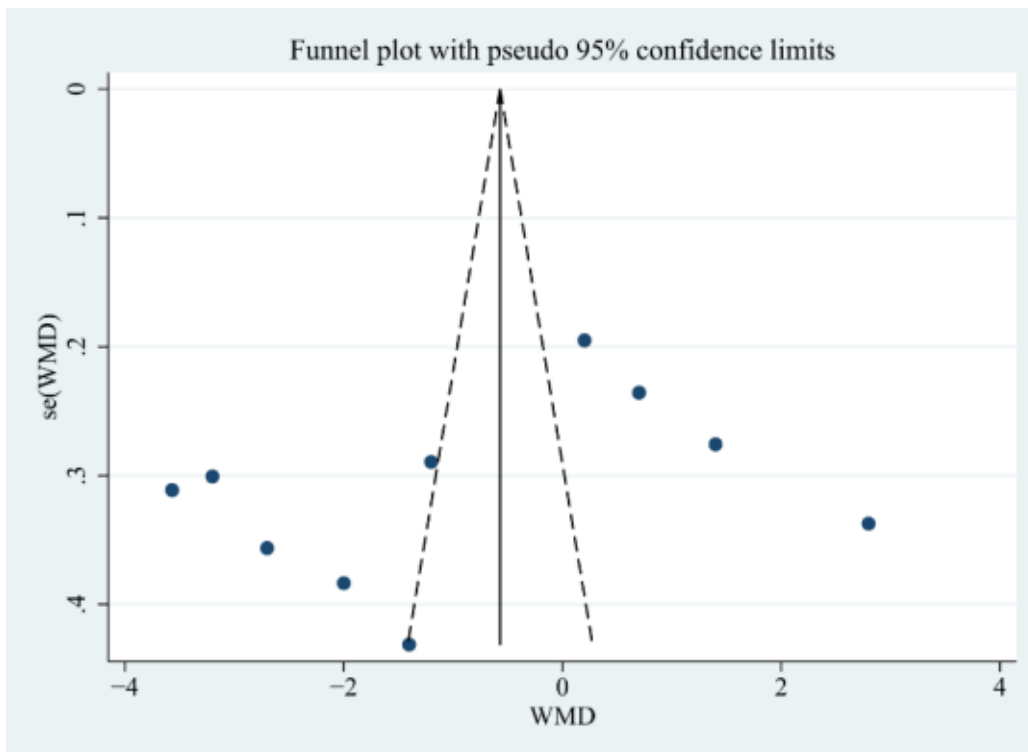

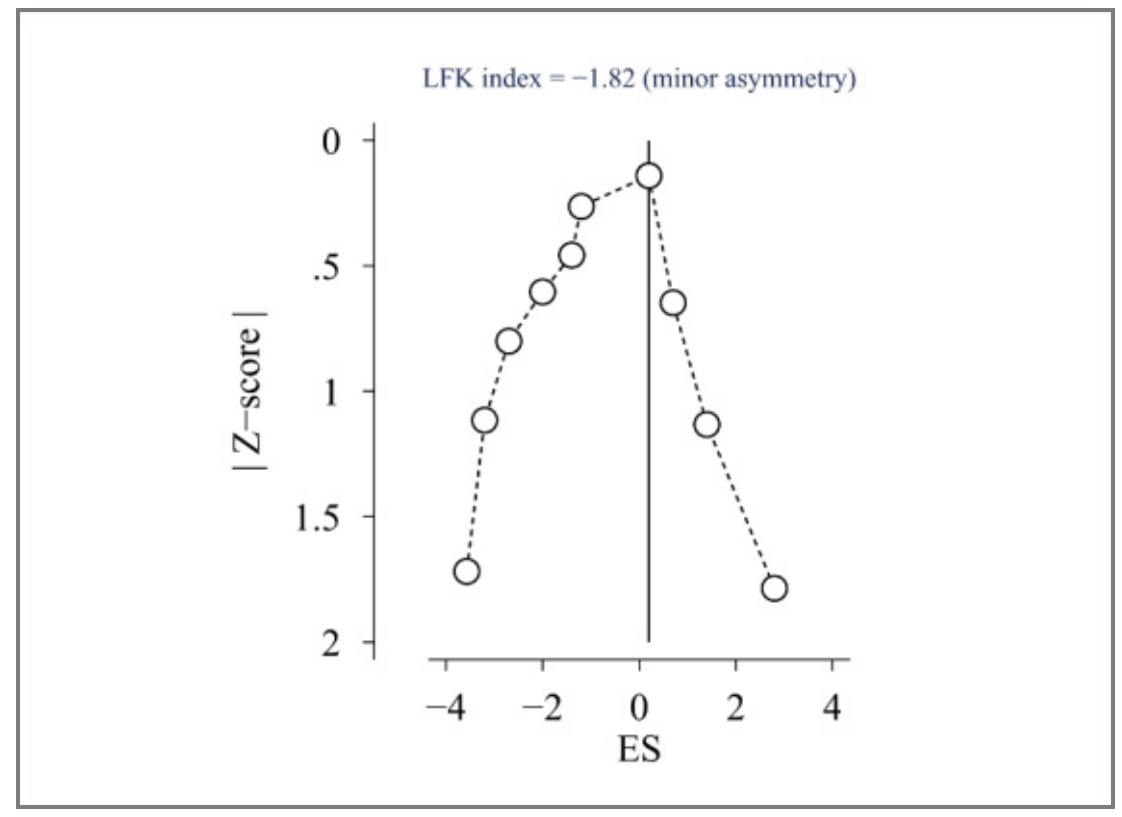

Fig. (S4). Funnel plot and Doi plot of studies evaluating the effect of IDegLira on body weight: (**a)** Funnel plot. (**b)** Doi plot. WMD: weighted mean difference, se: standard error, ES: effect size.

(A)

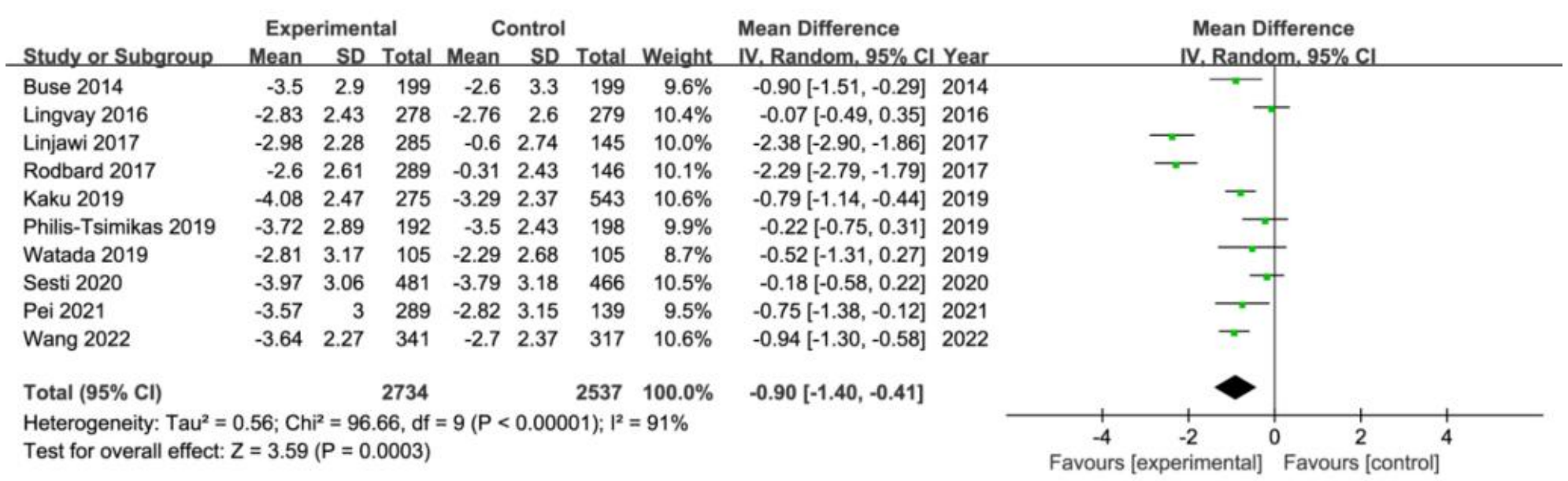

Study or Subgroup	Experimental Mean	Experimental SD	Experimental Total	Control Mean	Control SD	Control Total	Weight	Mean Difference IV, Random, 95% CI	Year
Buse 2014	-3.5	2.9	199	-2.6	3.3	199	9.6%	-0.90 [-1.51, -0.29]	2014
Lingvay 2016	-2.83	2.43	278	-2.76	2.6	279	10.4%	-0.07 [-0.49, 0.35]	2016
Linjawi 2017	-2.98	2.28	285	-0.6	2.74	145	10.0%	-2.38 [-2.90, -1.86]	2017
Rodbard 2017	-2.6	2.61	289	-0.31	2.43	146	10.1%	-2.29 [-2.79, -1.79]	2017
Kaku 2019	-4.08	2.47	275	-3.29	2.37	543	10.6%	-0.79 [-1.14, -0.44]	2019
Philis-Tsimikas 2019	-3.72	2.89	192	-3.5	2.43	198	9.9%	-0.22 [-0.75, 0.31]	2019
Watada 2019	-2.81	3.17	105	-2.29	2.68	105	8.7%	-0.52 [-1.31, 0.27]	2019
Sesti 2020	-3.97	3.06	481	-3.79	3.18	466	10.5%	-0.18 [-0.58, 0.22]	2020
Pei 2021	-3.57	3	289	-2.82	3.15	139	9.5%	-0.75 [-1.38, -0.12]	2021
Wang 2022	-3.64	2.27	341	-2.7	2.37	317	10.6%	-0.94 [-1.30, -0.58]	2022
**Total (95% CI)**			**2734**			**2537**	**100.0%**	**-0.90 [-1.40, -0.41]**	

Heterogeneity: Tau² = 0.56; Chi² = 96.66, df = 9 (P < 0.00001); I² = 91%
Test for overall effect: Z = 3.59 (P = 0.0003)

(B)

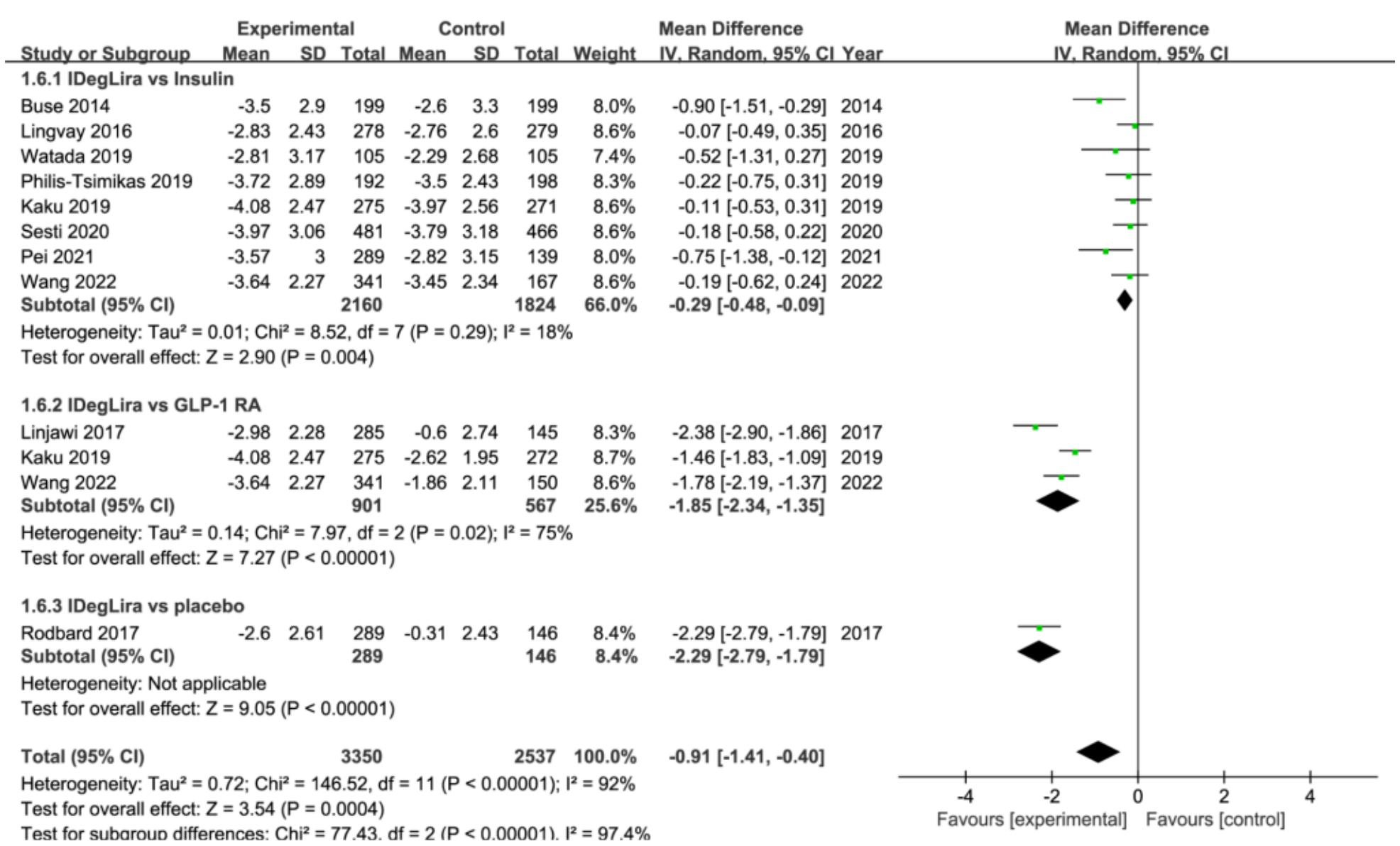

Study or Subgroup	Experimental Mean	Experimental SD	Experimental Total	Control Mean	Control SD	Control Total	Weight	Mean Difference IV, Random, 95% CI	Year
**1.6.1 IDegLira vs Insulin**									
Buse 2014	-3.5	2.9	199	-2.6	3.3	199	8.0%	-0.90 [-1.51, -0.29]	2014
Lingvay 2016	-2.83	2.43	278	-2.76	2.6	279	8.6%	-0.07 [-0.49, 0.35]	2016
Watada 2019	-2.81	3.17	105	-2.29	2.68	105	7.4%	-0.52 [-1.31, 0.27]	2019
Philis-Tsimikas 2019	-3.72	2.89	192	-3.5	2.43	198	8.3%	-0.22 [-0.75, 0.31]	2019
Kaku 2019	-4.08	2.47	275	-3.97	2.56	271	8.6%	-0.11 [-0.53, 0.31]	2019
Sesti 2020	-3.97	3.06	481	-3.79	3.18	466	8.6%	-0.18 [-0.58, 0.22]	2020
Pei 2021	-3.57	3	289	-2.82	3.15	139	8.0%	-0.75 [-1.38, -0.12]	2021
Wang 2022	-3.64	2.27	341	-3.45	2.34	167	8.6%	-0.19 [-0.62, 0.24]	2022
**Subtotal (95% CI)**			**2160**			**1824**	**66.0%**	**-0.29 [-0.48, -0.09]**	
Heterogeneity: Tau² = 0.01; Chi² = 8.52, df = 7 (P = 0.29); I² = 18%									
Test for overall effect: Z = 2.90 (P = 0.004)									
**1.6.2 IDegLira vs GLP-1 RA**									
Linjawi 2017	-2.98	2.28	285	-0.6	2.74	145	8.3%	-2.38 [-2.90, -1.86]	2017
Kaku 2019	-4.08	2.47	275	-2.62	1.95	272	8.7%	-1.46 [-1.83, -1.09]	2019
Wang 2022	-3.64	2.27	341	-1.86	2.11	150	8.6%	-1.78 [-2.19, -1.37]	2022
**Subtotal (95% CI)**			**901**			**567**	**25.6%**	**-1.85 [-2.34, -1.35]**	
Heterogeneity: Tau² = 0.14; Chi² = 7.97, df = 2 (P = 0.02); I² = 75%									
Test for overall effect: Z = 7.27 (P < 0.00001)									
**1.6.3 IDegLira vs placebo**									
Rodbard 2017	-2.6	2.61	289	-0.31	2.43	146	8.4%	-2.29 [-2.79, -1.79]	2017
**Subtotal (95% CI)**			**289**			**146**	**8.4%**	**-2.29 [-2.79, -1.79]**	
Heterogeneity: Not applicable									
Test for overall effect: Z = 9.05 (P < 0.00001)									
**Total (95% CI)**			**3350**			**2537**	**100.0%**	**-0.91 [-1.41, -0.40]**	

Heterogeneity: Tau² = 0.72; Chi² = 146.52, df = 11 (P < 0.00001); I² = 92%
Test for overall effect: Z = 3.54 (P = 0.0004)
Test for subgroup differences: Chi² = 77.43, df = 2 (P < 0.00001), I² = 97.4%

Fig. (S5). (**A**) Forest plot of studies evaluating the effect of IDegLira in FPG (mmol/L). (**B**) Forest plot of the subgroup analysis by control types for evaluating the effect of IDegLira in FPG (mmol/L). Effect sizes: mean difference (MD), heterogeneity metrics: I², error bars represent 95% confidence intervals (CIs).

(a)

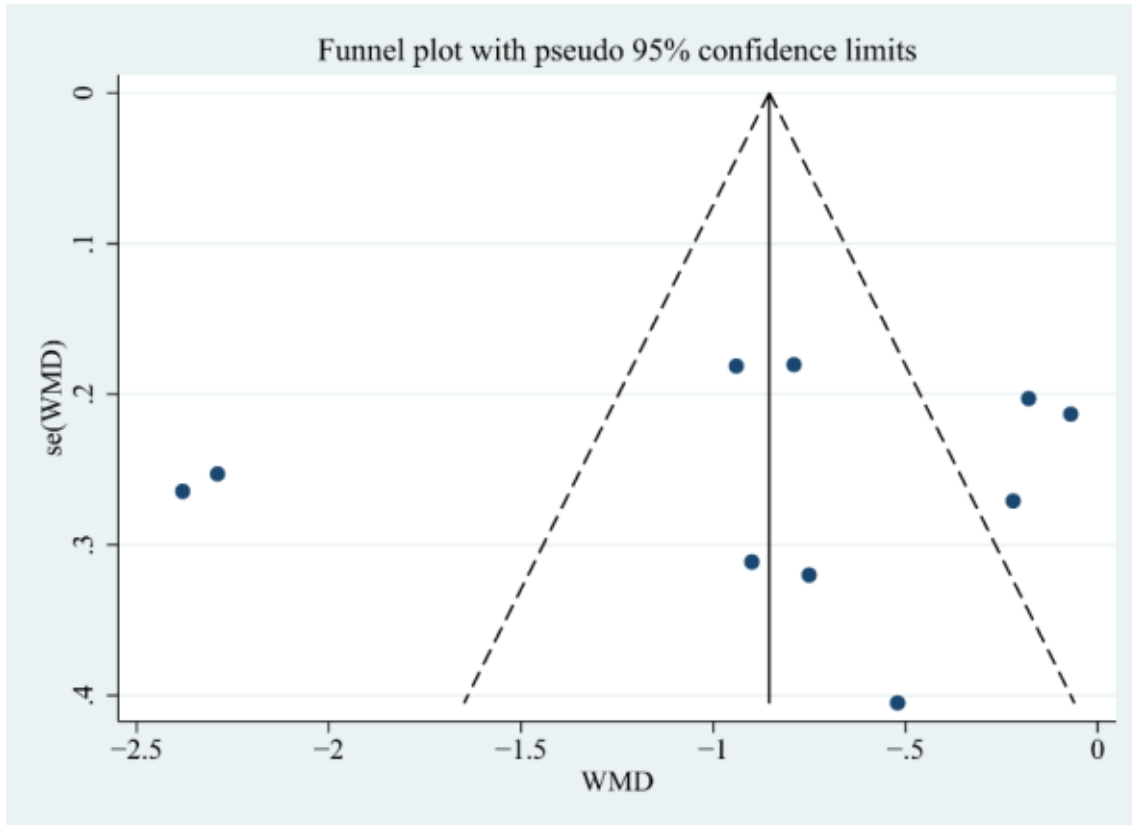

(b)

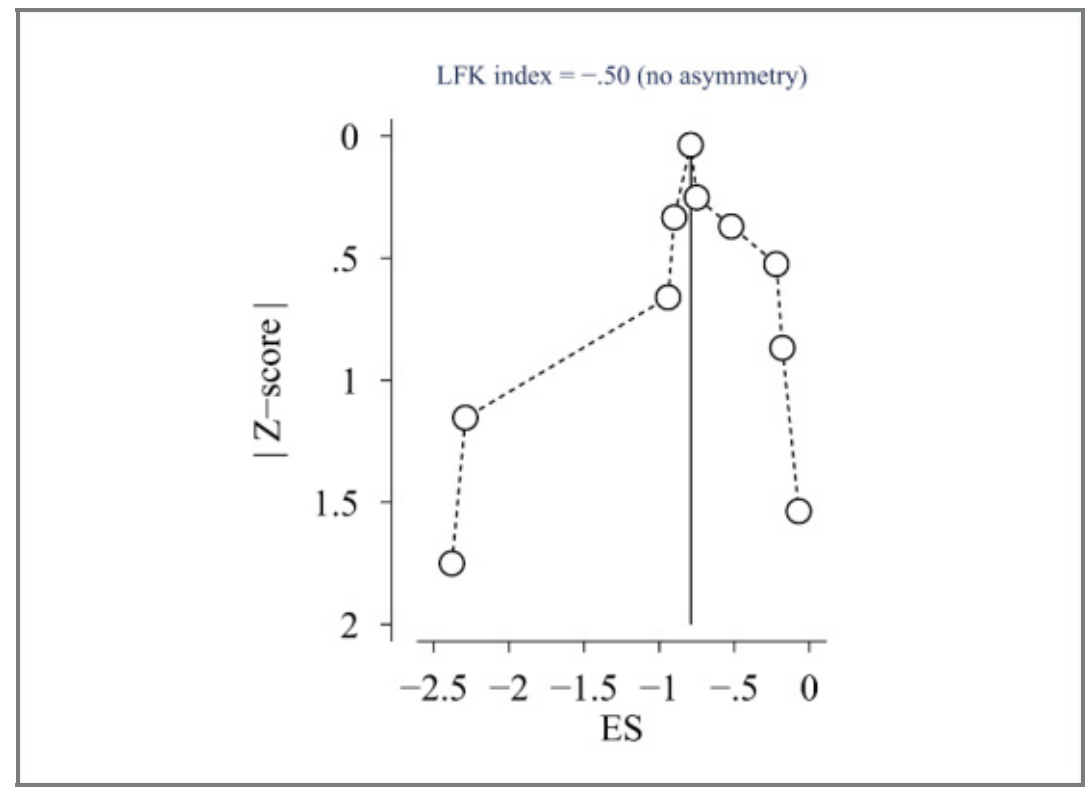

Fig. (S6). Funnel plot and Doi plot of studies evaluating the effect of IDegLira on FPG: (**a**) Funnel plot. (**b**) Doi plot. WMD: weighted mean difference, se: standard error, ES: effect size.

(A)

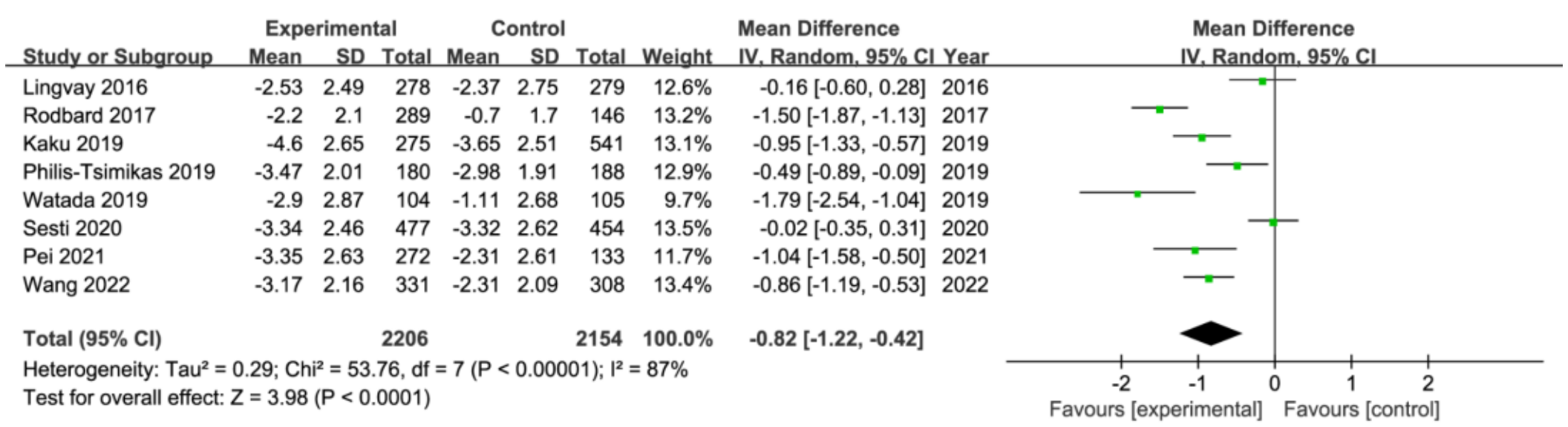

(B)

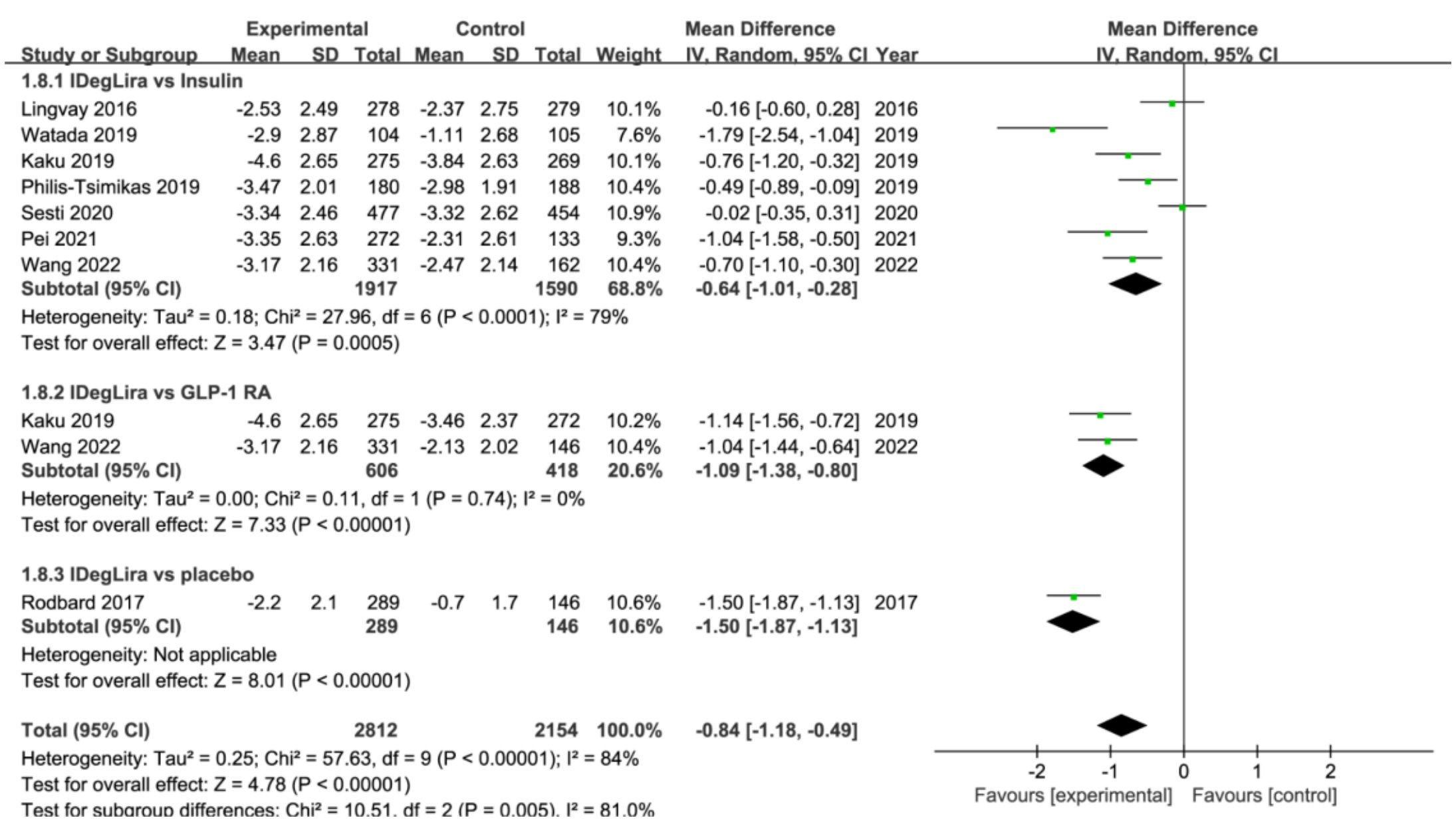

Fig. (S7). (**A**) Forest plot of studies evaluating the effect of IDegLira in SMPG (mmol/L) (the mean of the nine-point SMPG). (**B**) Forest plot of the subgroup analysis by control types for evaluating the effect of IDegLira in SMPG (mmol/L) (the mean of the nine-point SMPG). Effect sizes: mean difference (MD), heterogeneity metrics: I², error bars represent 95% confidence intervals (CIs).

(a) **(b)**

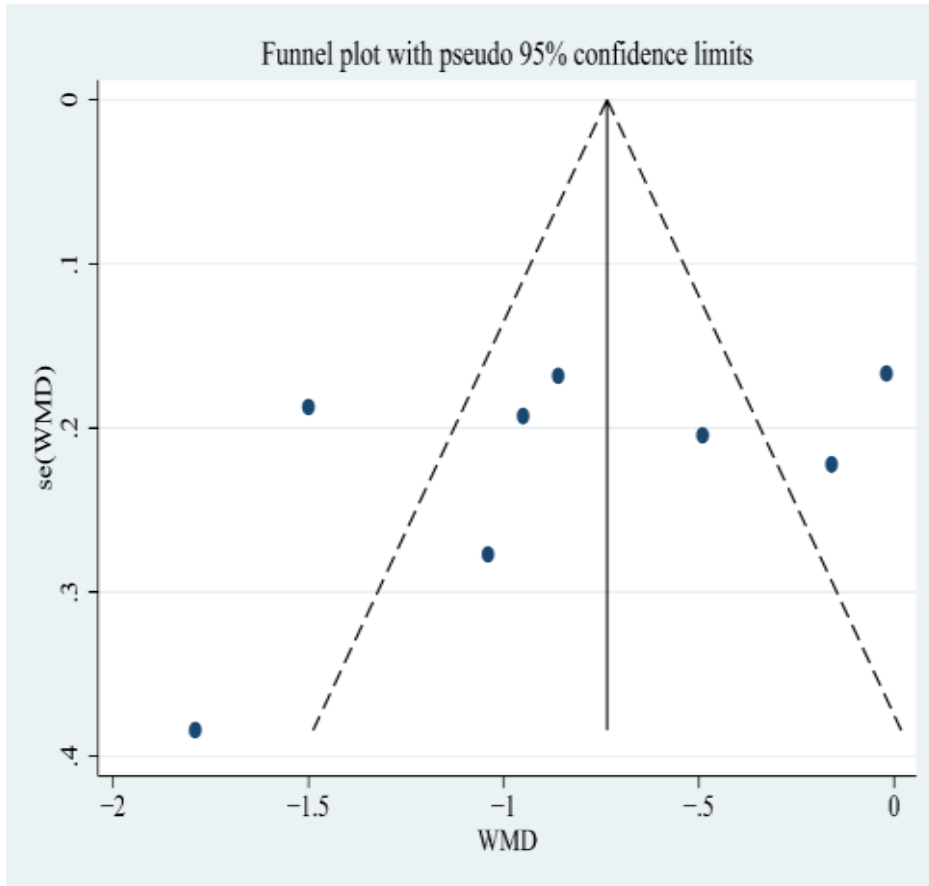

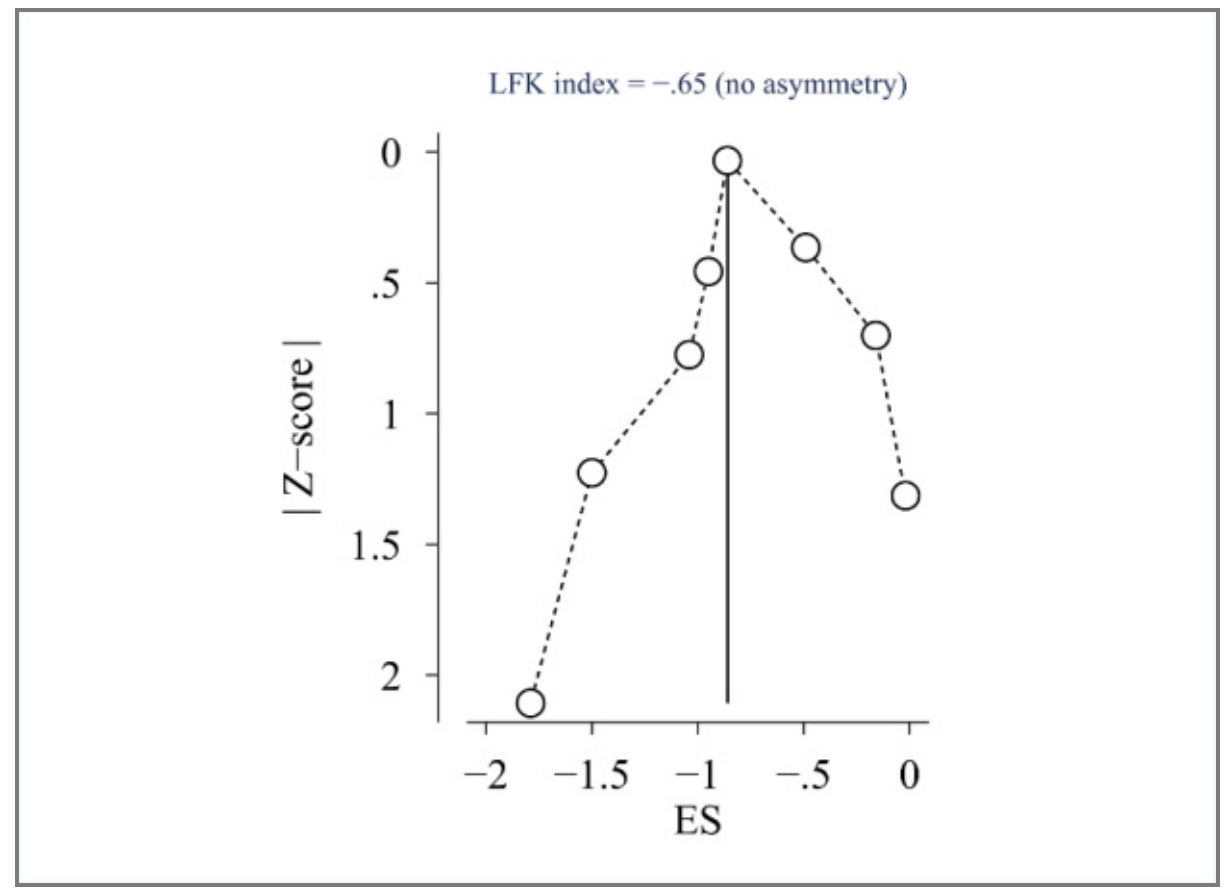

Fig. (S8). Funnel plot and Doi plot of studies evaluating the effect of IDegLira on SMPG: (**a**) Funnel plot. (**b)** Doi plot. WMD: weighted mean difference, se: standard error, ES: effect size.

(A)

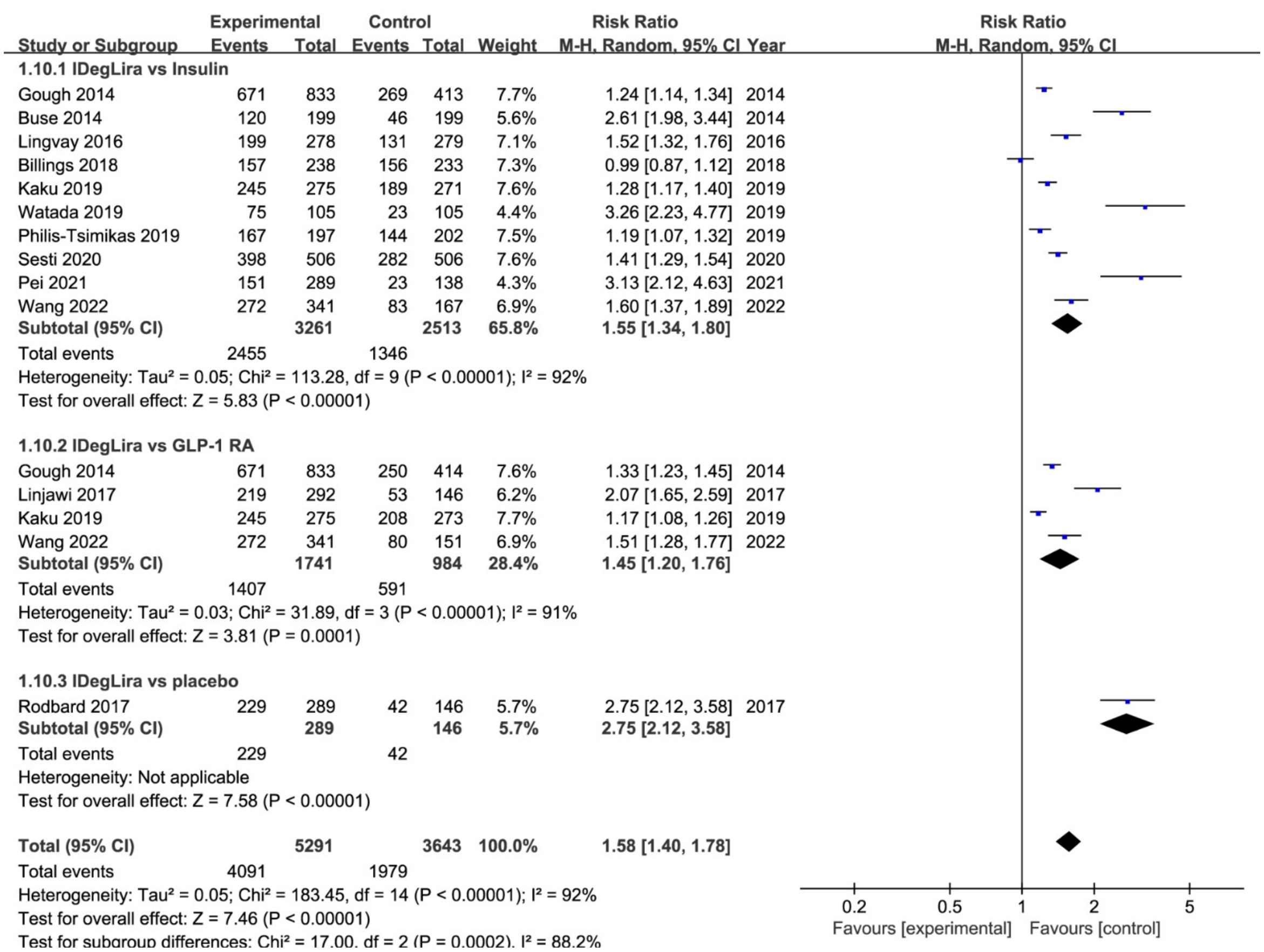

(B)

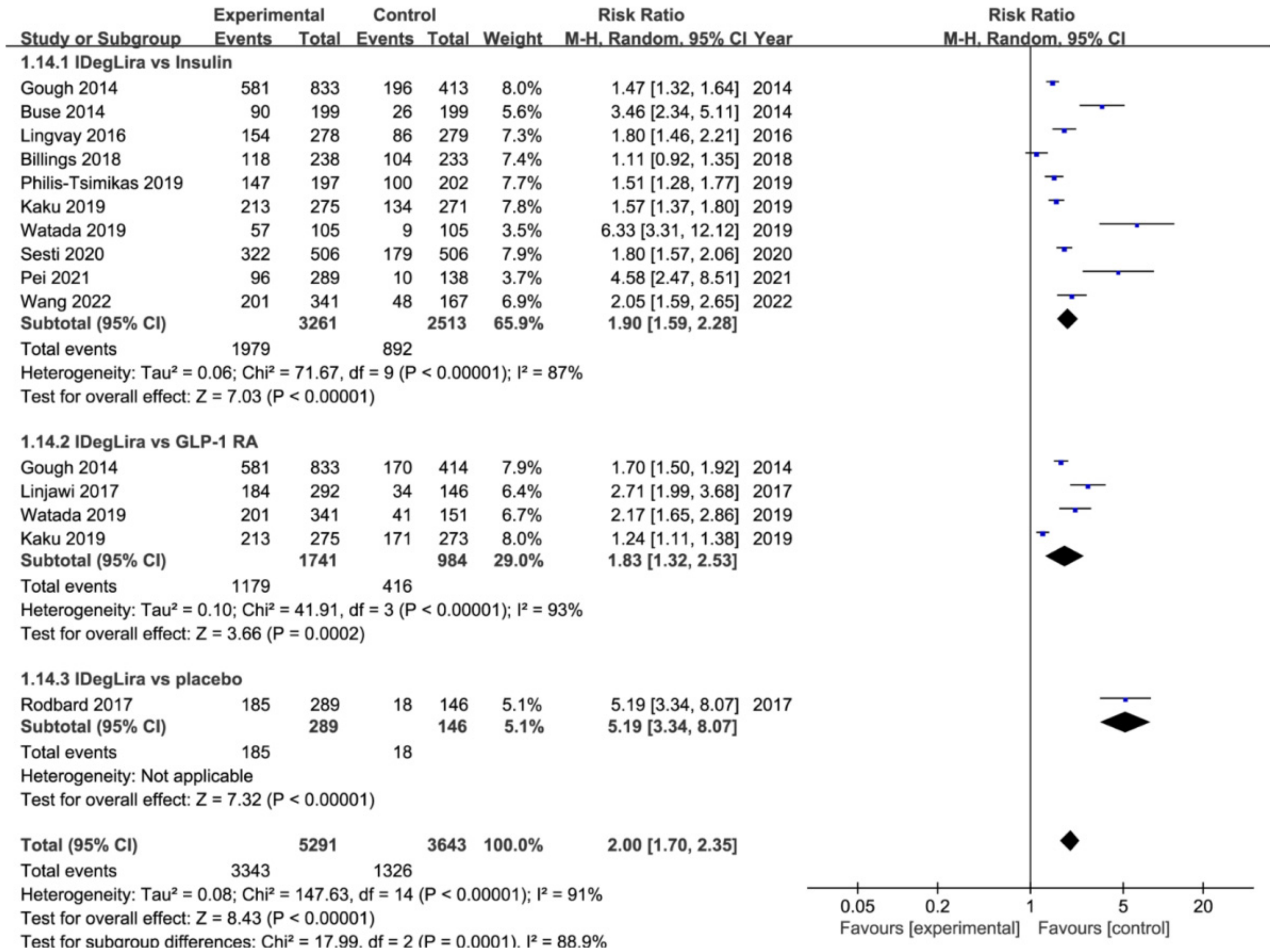

Fig. (S6). (**A**) Forest plot of the subgroup analysis by control types for evaluating the effect of IDegLira in achieving the target of HbA1c < 7.0%. (**B**) Forest plot of the subgroup analysis by control types for evaluating the effect of IDegLira in achieving the target of HbA1c < 6.5%. Effect sizes: risk ratio (RR), heterogeneity metrics: I², error bars represent 95% confidence intervals (CIs).

(a)

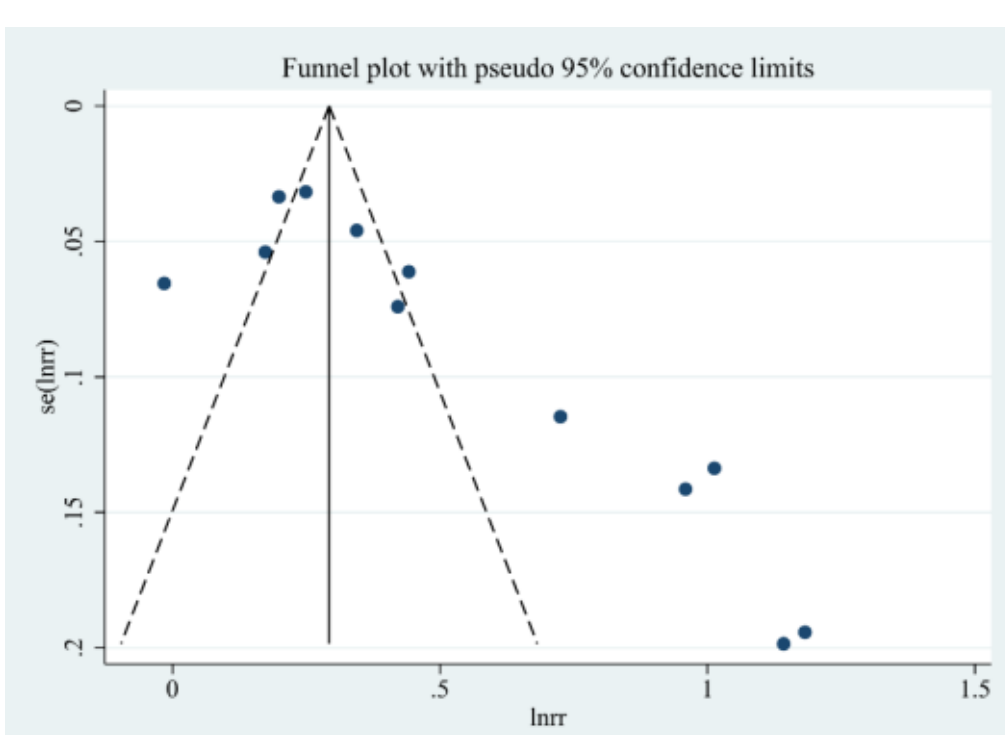

(b)

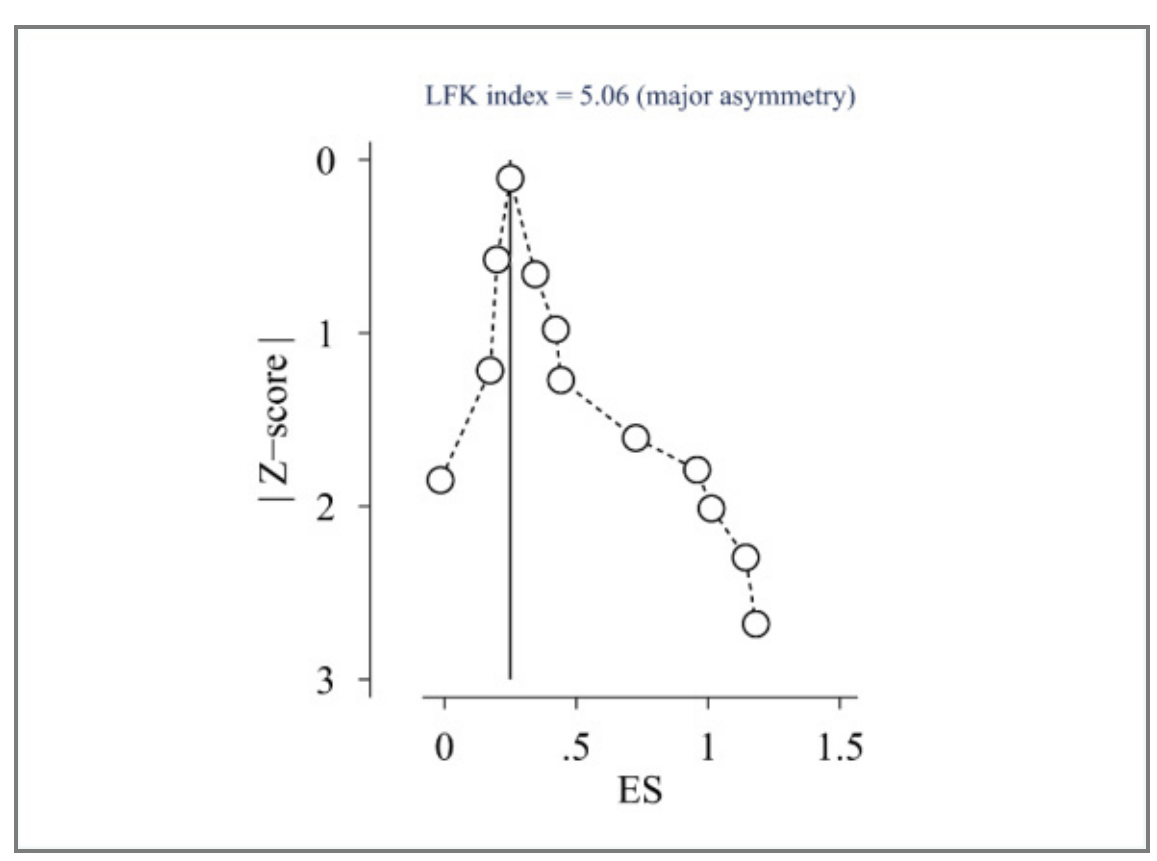

Fig. (S10). Funnel plot and Doi plot of studies evaluating the effect of IDegLira on achieving the target of HbA1c <7.0%: (**a**) Funnel plot. (**b**) Doi plot. WMD: weighted mean difference, se: standard error, ES: effect size.

(a) **(b)**

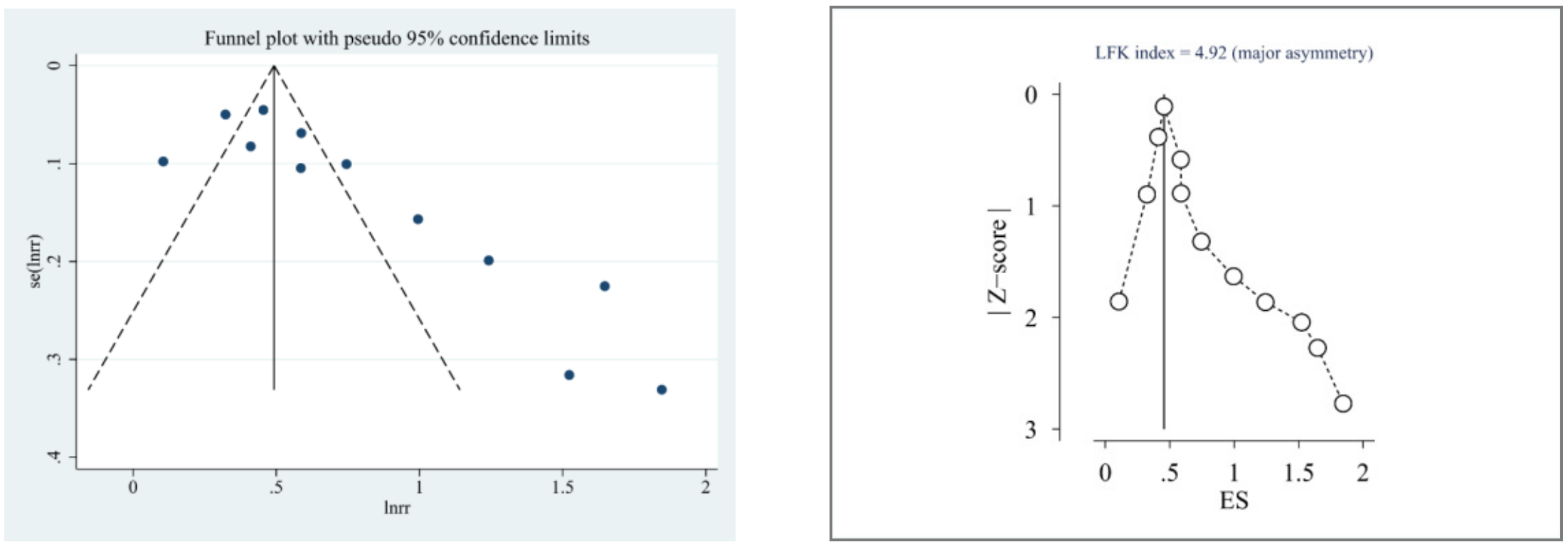

Fig. (S11). Funnel plot and Doi plot of studies evaluating the effect of IDegLira on achieving the target of HbA1c <6.5%: (**a**) Funnel plot. (**b**) Doi plot. WMD: weighted mean difference, se: standard error, ES: effect size.

(A)

```
. meta trimfill, estimator(linear) funnel eform

  Effect-size label: Log risk-ratio
        Effect size: _meta_es
          Std. err.: _meta_se

Nonparametric trim-and-fill analysis of publication bias
Linear estimator, imputing on the left

Iteration                            Number of studies =     14
  Model: Random-effects                       observed =     12
 Method: DerSimonian-Laird                    imputed =      2

Pooling
  Model: Random-effects
 Method: DerSimonian-Laird
```

Studies	Risk ratio	[95% conf.	interval]
Observed	1.650	1.441	1.889
Observed + Imputed	1.520	1.329	1.738

(B)

```
. meta trimfill, estimator(linear) funnel eform

  Effect-size label: Log risk-ratio
        Effect size: _meta_es
          Std. err.: _meta_se

Nonparametric trim-and-fill analysis of publication bias
Linear estimator, imputing on the left

Iteration                            Number of studies =     12
  Model: Random-effects                       observed =     12
 Method: REML                                  imputed =      0

Pooling
  Model: Random-effects
 Method: REML
```

Studies	Risk ratio	[95% conf.	interval]
Observed	2.263	1.678	3.053
Observed + Imputed	2.263	1.678	3.053

Fig. (S12). The result of non-parametric trim and fill analysis of the pooled proportion of participants achieving the HbA1c level of < 7.0% (**A**) or < 6.5% (**B**) using the random-effect analysis.

(a) **(b)**

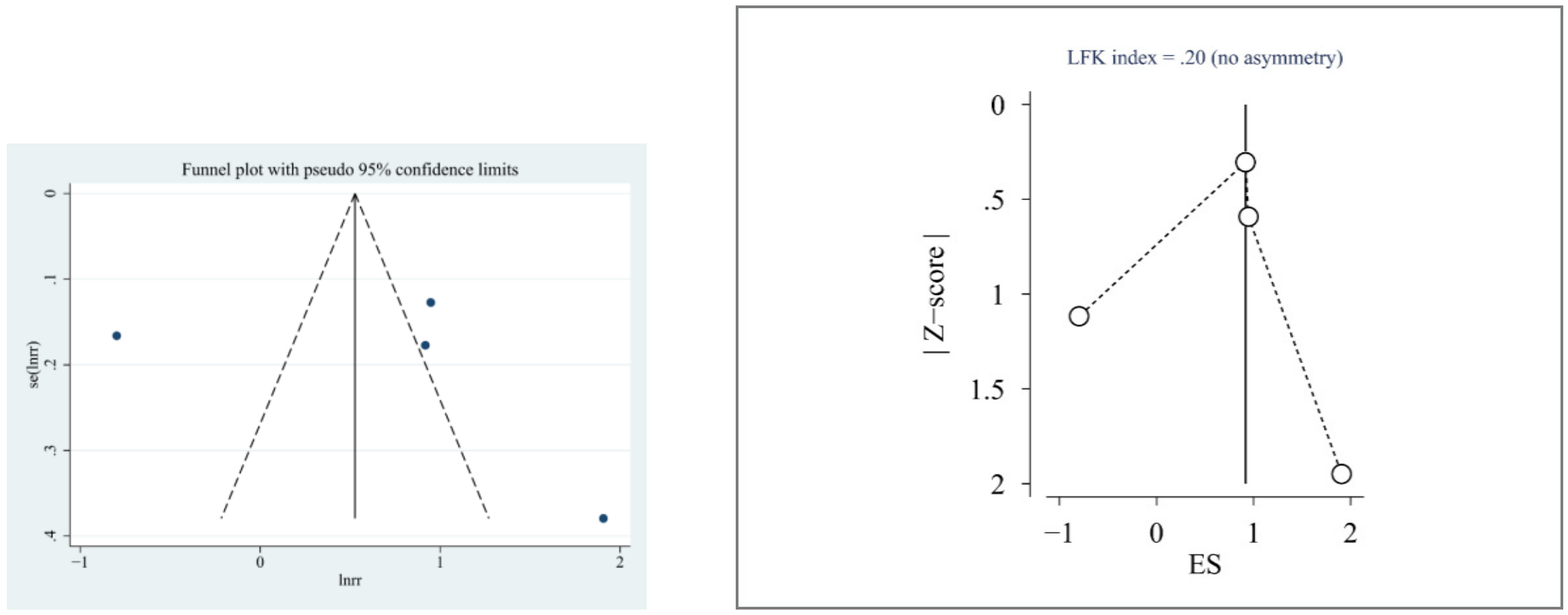

Fig. (S13). Funnel plot and Doi plot of studies evaluating the effect of IDegLira on achieving the target of HbA1c <7.0% without weight gain and hypoglycemia: (**a**) Funnel plot. (**b**) Doi plot. WMD: weighted mean difference, se: standard error, ES: effect size.

(a) **(b)**

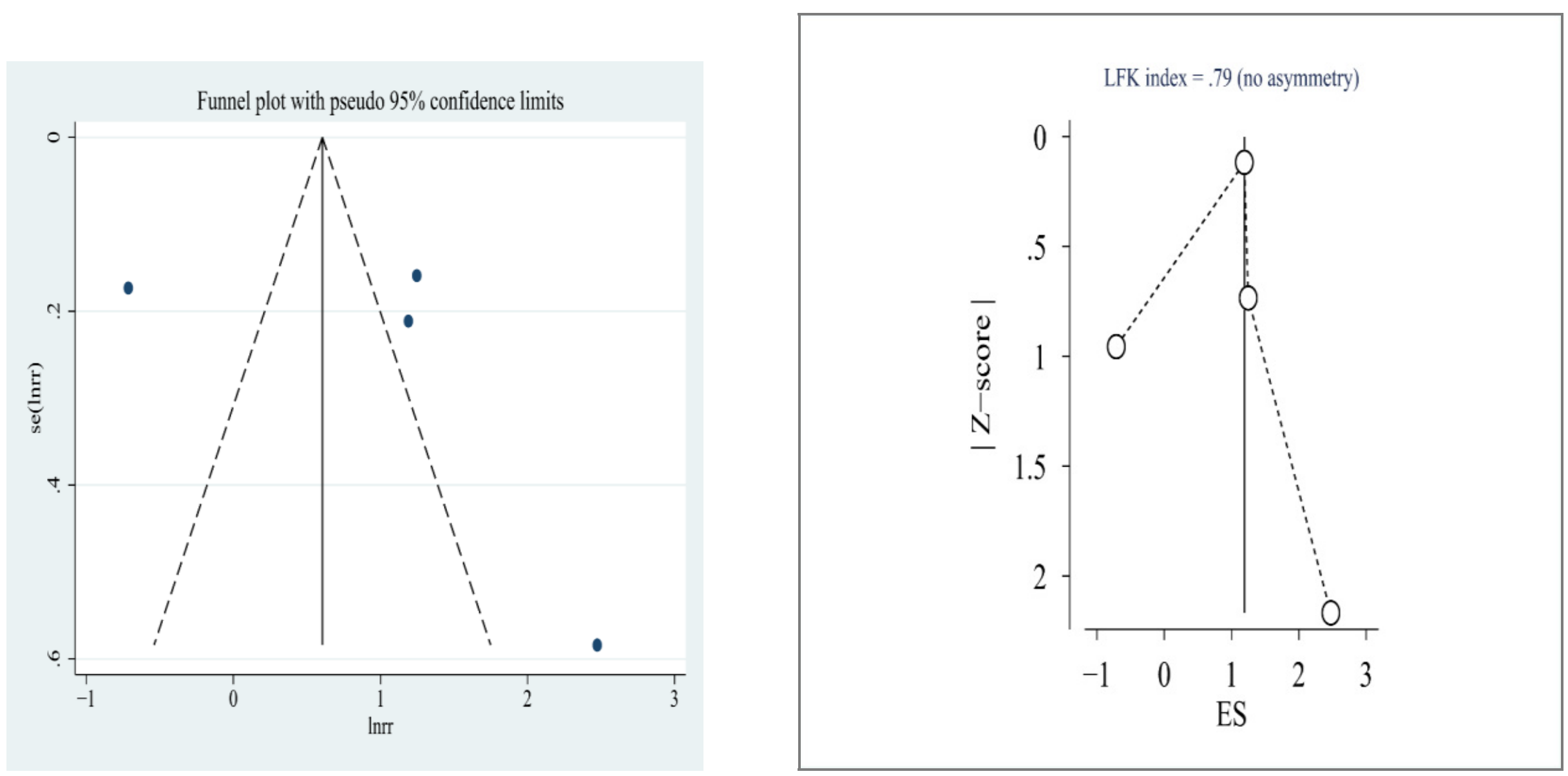

Fig. (S14). Funnel plot and Doi plot of studies evaluating the effect of IDegLira on achieving the target of HbA1c <6.5% without weight gain and hypoglycemia: (**a**) Funnel plot. (**b**) Doi plot. WMD: weighted mean difference, se: standard error, ES: effect size.

(A)

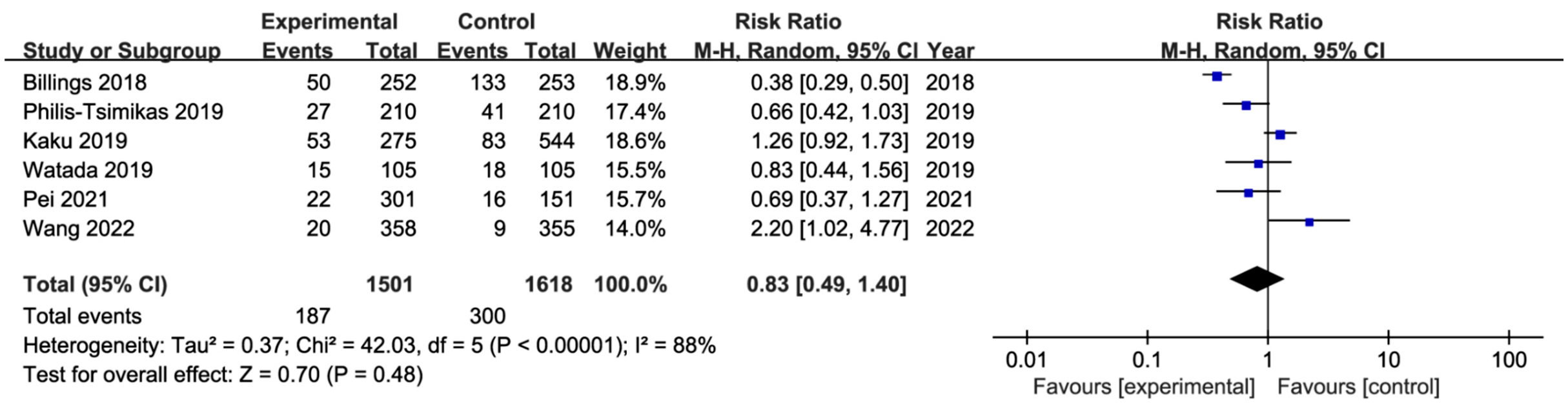

(B)

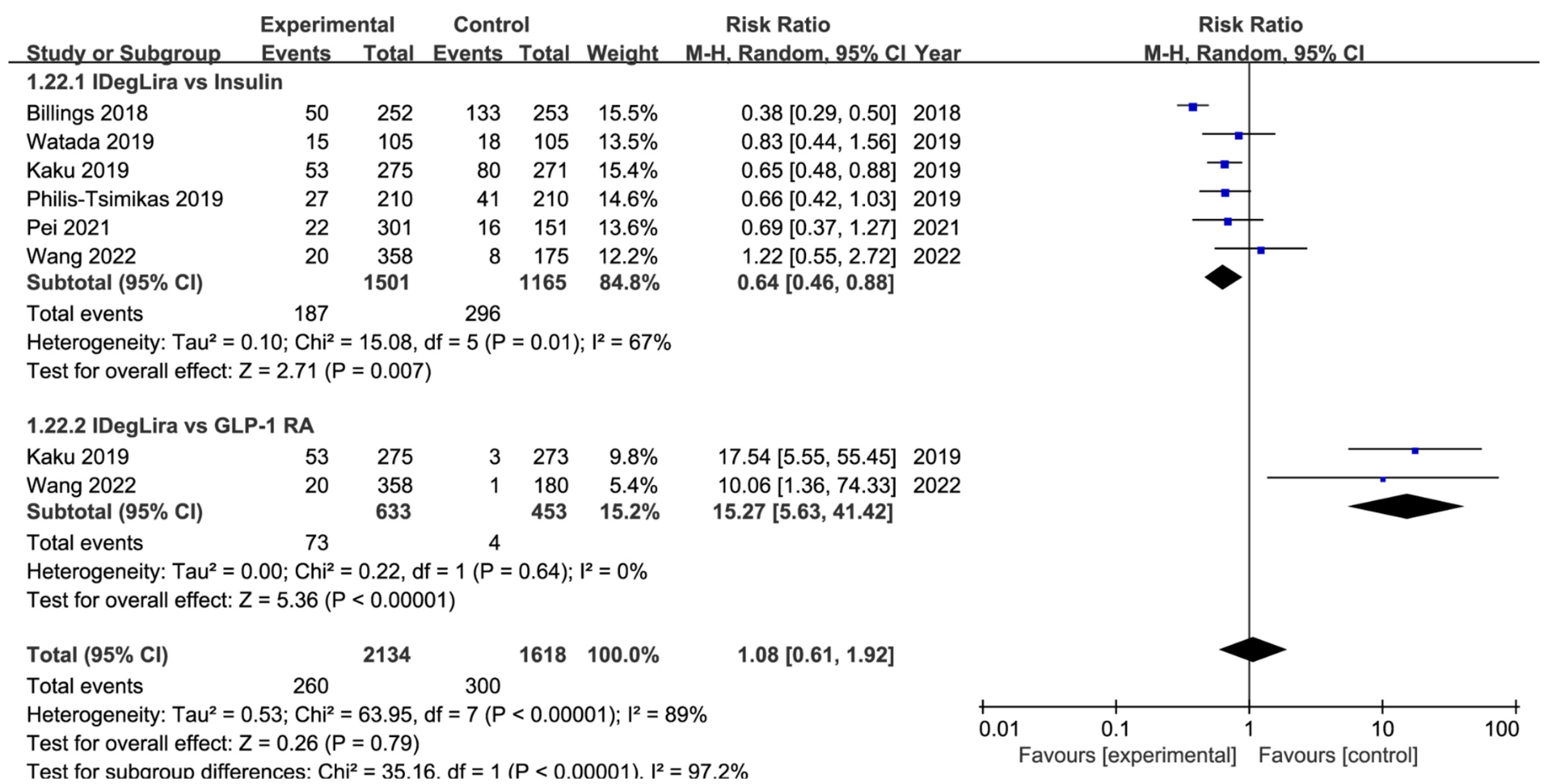

Fig. (S15). (**A**) Forest plot of studies evaluating the effect of IDegLira in hypoglycemia (severe or BG-confirmed symptomatic). (**B**) Forest plot of the subgroup analysis by control types for evaluating the effect of IDegLira in hypoglycemia (severe or BG-confirmed symptomatic). Effect sizes: risk ratio (RR), heterogeneity metrics: I², error bars represent 95% confidence intervals (CIs).

(a)

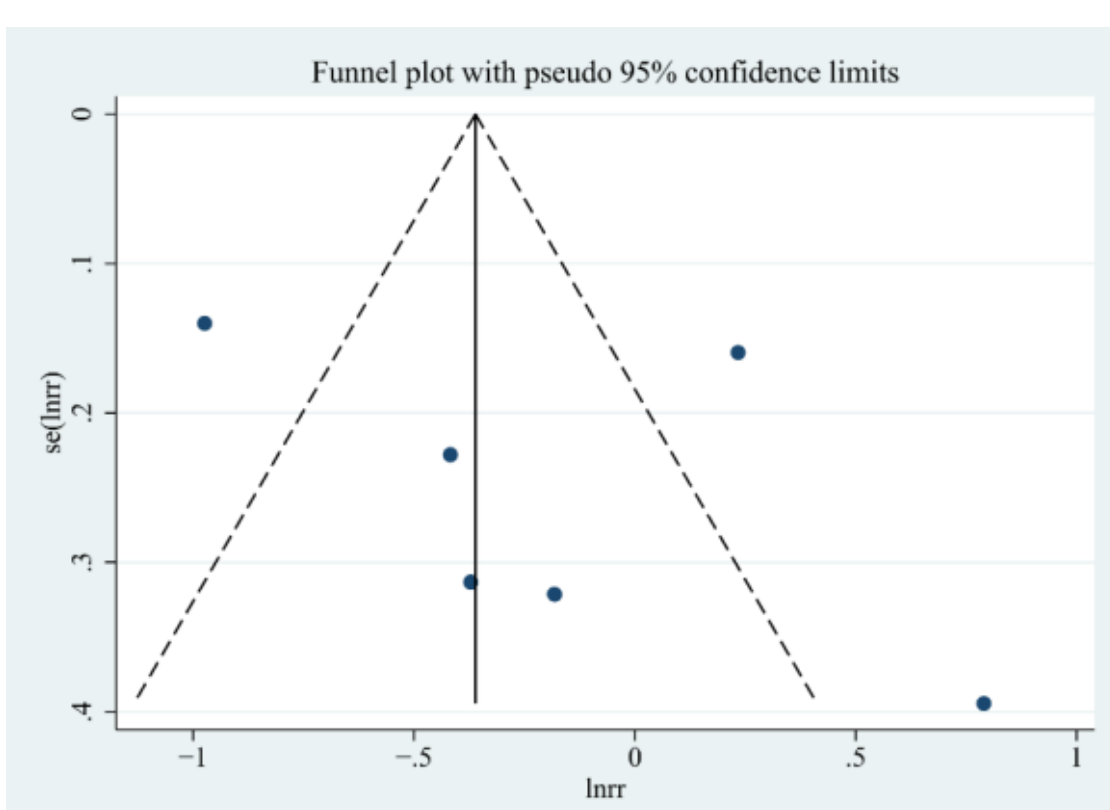

(b)

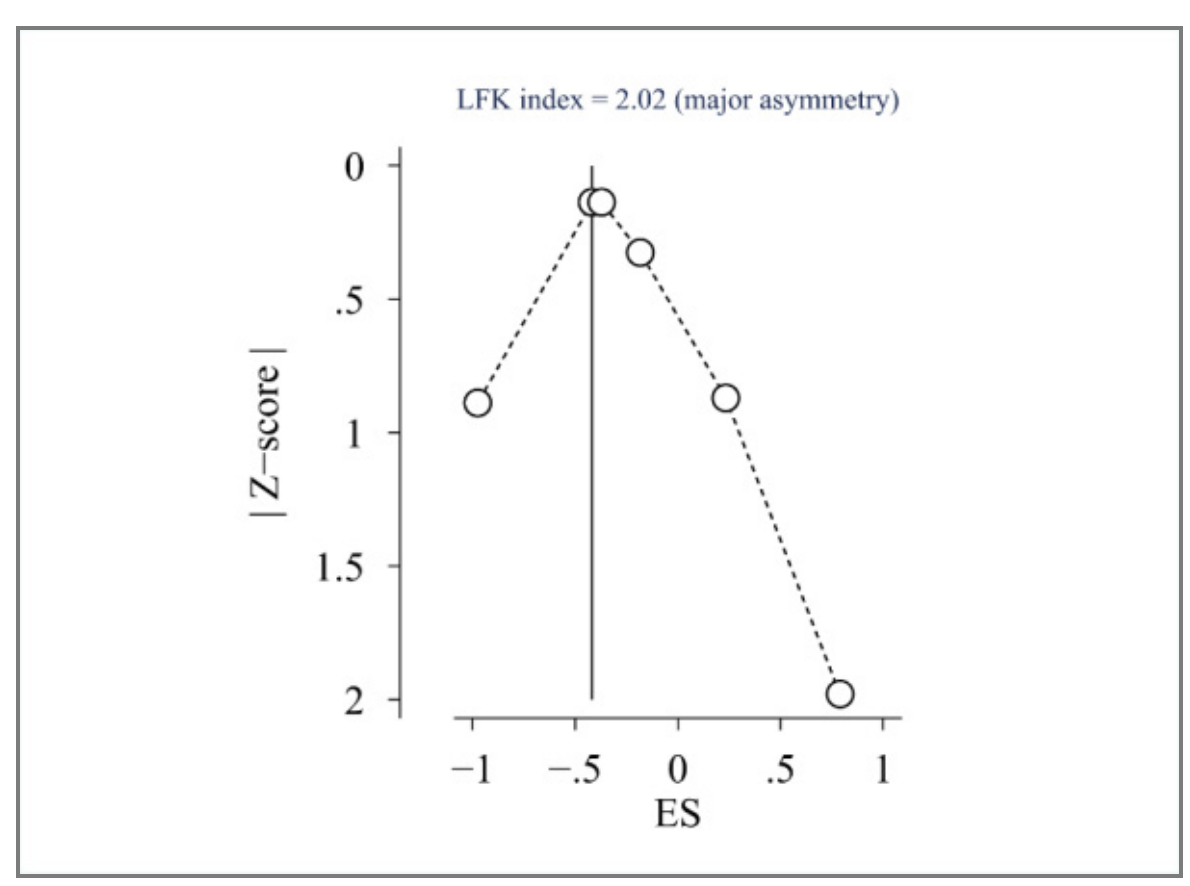

Fig. (S16). Funnel plot and Doi plot of studies evaluating the effect of IDegLira on hypoglycemic events: (**a**) Funnel plot. (**b**) Doi plot. WMD: weighted mean difference, se: standard error, ES: effect size.

```
. meta trimfill, estimator(linear) funnel eform

  Effect-size label: Log risk-ratio
        Effect size: _meta_es
          Std. err.: _meta_se

Nonparametric trim-and-fill analysis of publication bias
Linear estimator, imputing on the left

Iteration                            Number of studies =      7
  Model: Random-effects                       observed =      7
 Method: REML                                 imputed =      0

Pooling
  Model: Random-effects
 Method: REML

----------------------------------------------------------------
             Studies |  Risk ratio    [95% conf. interval]
---------------------+------------------------------------------
            Observed |       0.697       0.589       0.825
 Observed + Imputed  |       0.697       0.589       0.825
----------------------------------------------------------------
```

Fig. (S17). The result of non-parametric trim and fill analysis of the pooled incidence of hypoglycemia using the random-effect analysis.

(A)

Study or Subgroup	Experimental Events	Experimental Total	Control Events	Control Total	Weight	Risk Ratio M-H, Random, 95% CI	Year
Buse 2014	115	199	122	199	4.3%	0.94 [0.80, 1.11]	2014
Gough 2014	521	825	547	824	22.1%	0.95 [0.89, 1.02]	2014
Linjawi 2017	191	291	92	145	5.1%	1.03 [0.89, 1.20]	2017
Rodbard 2017	185	288	85	146	4.3%	1.10 [0.94, 1.30]	2017
Billings 2018	149	252	144	253	5.1%	1.04 [0.90, 1.21]	2018
Philis-Tsimikas 2019	129	209	123	210	4.6%	1.05 [0.90, 1.23]	2019
Kaku 2019	229	275	445	544	25.6%	1.02 [0.95, 1.09]	2019
Watada 2019	82	105	80	105	5.2%	1.02 [0.88, 1.19]	2019
Pei 2021	216	301	110	151	7.7%	0.99 [0.87, 1.11]	2021
Wang 2022	267	358	260	335	16.1%	0.96 [0.88, 1.04]	2022
**Total (95% CI)**		**3103**		**2912**	**100.0%**	**0.99 [0.96, 1.03]**	
Total events	2084		2008				

Heterogeneity: Tau² = 0.00; Chi² = 5.93, df = 9 (P = 0.75); I² = 0%
Test for overall effect: Z = 0.29 (P = 0.77)

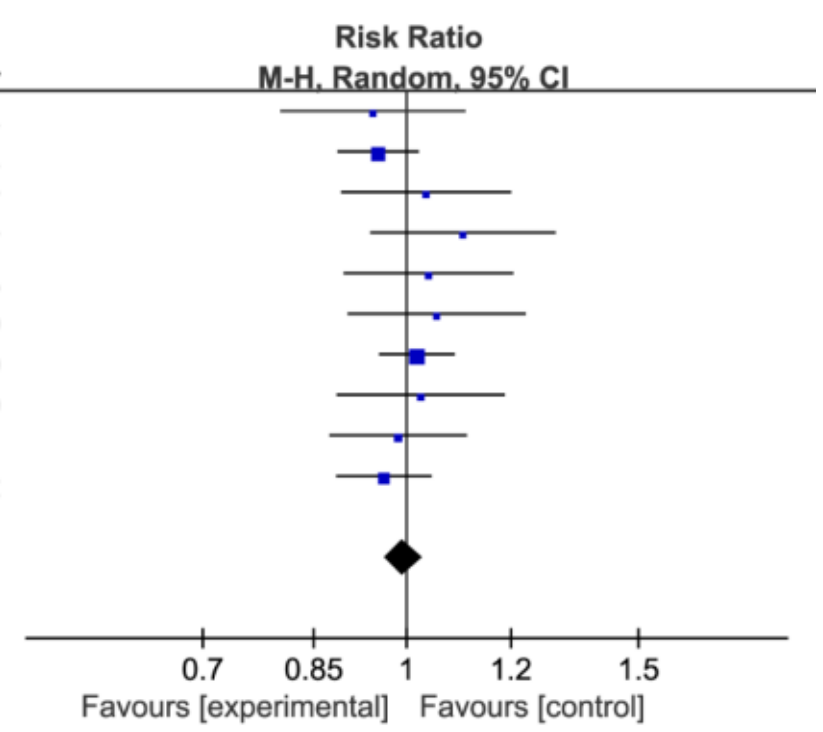

(B)

Study or Subgroup	Experimental Events	Experimental Total	Control Events	Control Total	Weight	Risk Ratio M-H, Random, 95% CI	Year
Buse 2014	7	199	11	199	7.5%	0.64 [0.25, 1.61]	2014
Gough 2014	19	825	22	824	17.5%	0.86 [0.47, 1.58]	2014
Lingvay 2016	5	278	9	279	5.5%	0.56 [0.19, 1.64]	2016
Rodbard 2017	14	288	5	146	6.4%	1.42 [0.52, 3.86]	2017
Linjawi 2017	9	291	3	145	3.9%	1.49 [0.41, 5.44]	2017
Billings 2018	12	252	10	253	9.5%	1.20 [0.53, 2.74]	2018
Philis-Tsimikas 2019	6	209	7	210	5.6%	0.86 [0.29, 2.52]	2019
Kaku 2019	17	275	27	544	18.5%	1.25 [0.69, 2.25]	2019
Watada 2019	3	105	4	105	3.0%	0.75 [0.17, 3.27]	2019
Pei 2021	13	301	7	151	8.0%	0.93 [0.38, 2.29]	2021
Wang 2022	14	358	21	355	14.7%	0.66 [0.34, 1.28]	2022
**Total (95% CI)**		**3381**		**3211**	**100.0%**	**0.92 [0.72, 1.19]**	
Total events	119		126				

Heterogeneity: Tau² = 0.00; Chi² = 5.22, df = 10 (P = 0.88); I² = 0%
Test for overall effect: Z = 0.61 (P = 0.54)

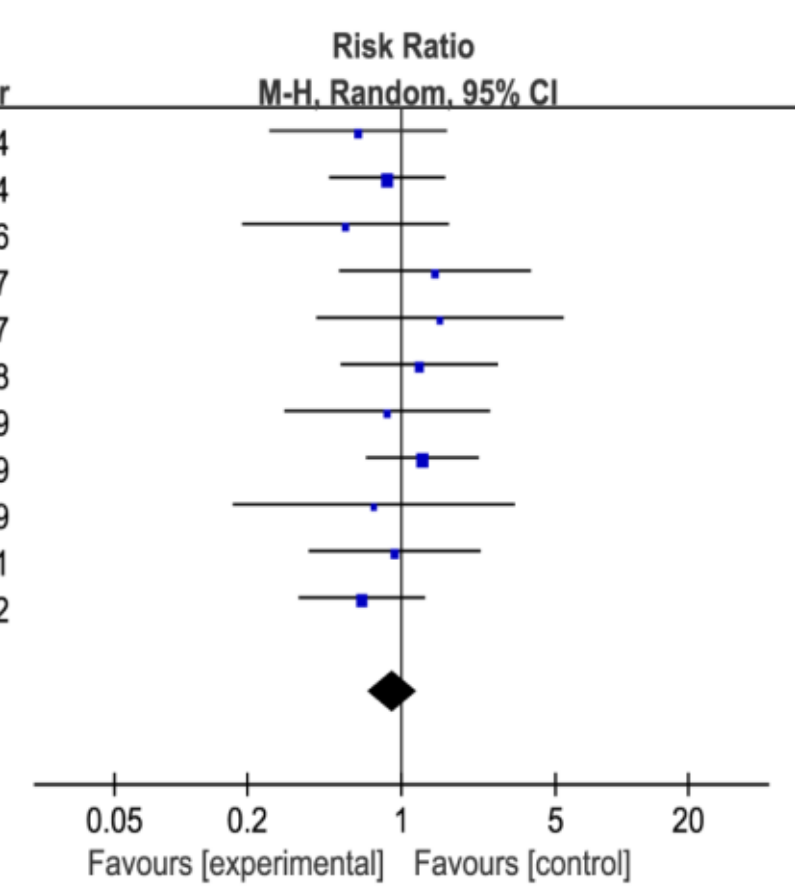

Fig. (S18). (**A**) Forest plot of studies evaluating the effect of IDegLira in adverse events. (**B**) Forest plot of studies evaluating the effect of IDegLira in serious adverse events. Effect sizes: risk ratio (RR), heterogeneity metrics: I², error bars represent 95% confidence intervals (CIs).

(A)

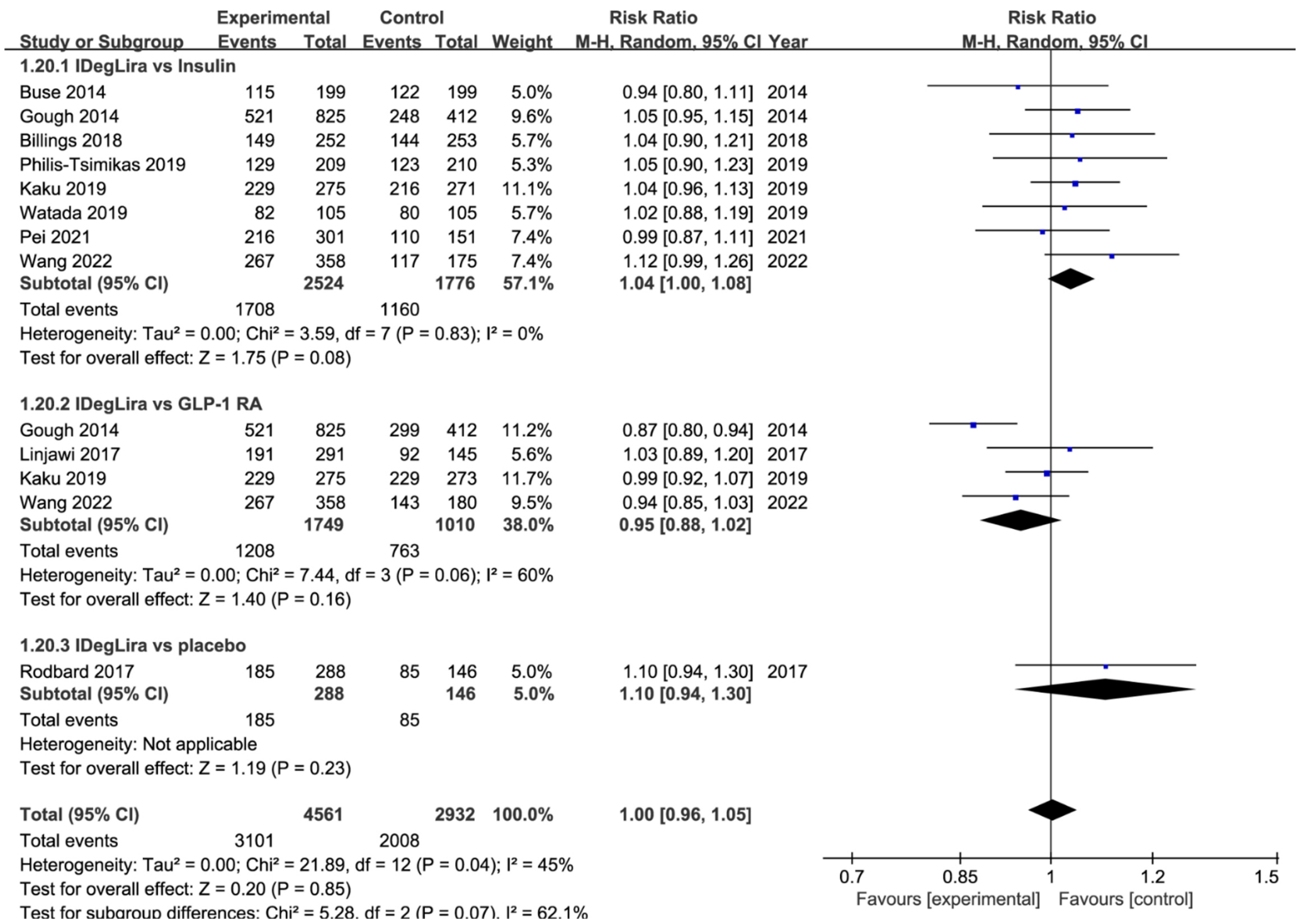

(B)

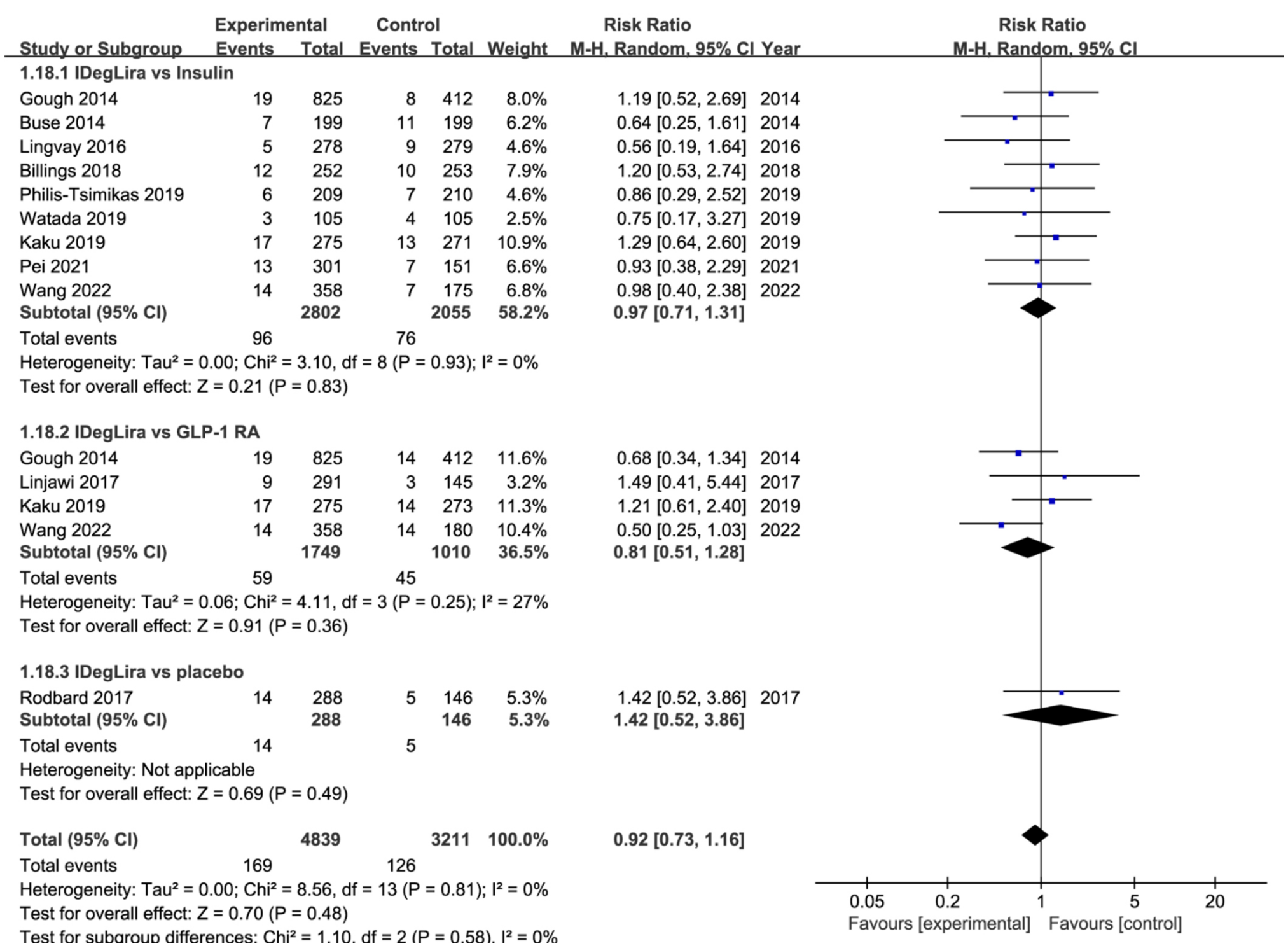

Fig. (S19). (**A**) Forest plot of the subgroup analysis by control types for evaluating the effect of IDegLira in adverse events. (**B**) Forest plot of the subgroup analysis by control types for evaluating the effect of IDegLira in serious adverse events. Effect sizes: risk ratio (RR), heterogeneity metrics: I², error bars represent 95% confidence intervals (CIs).

(a) **(b)**

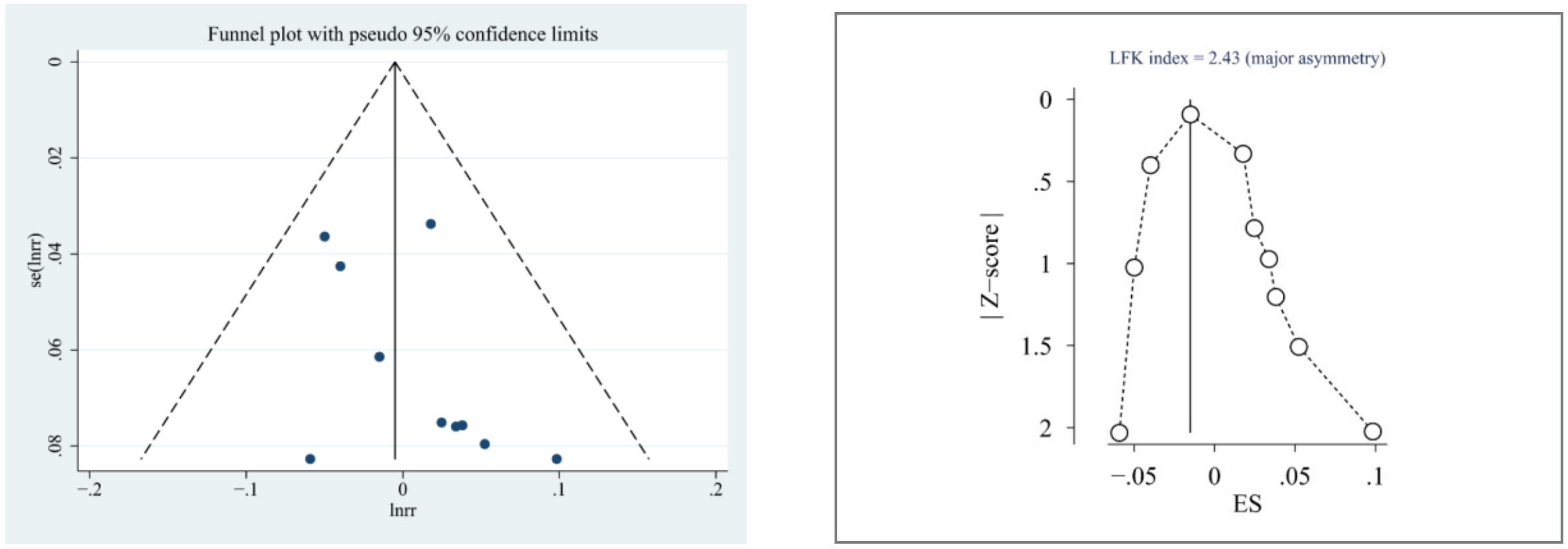

Fig. (S20). Funnel plot and Doi plot of studies evaluating the effect of IDegLira on adverse events: (**a)** Funnel plot. (**b)** Doi plot. WMD: weighted mean difference, se: standard error, ES: effect size.

(a) **(b)**

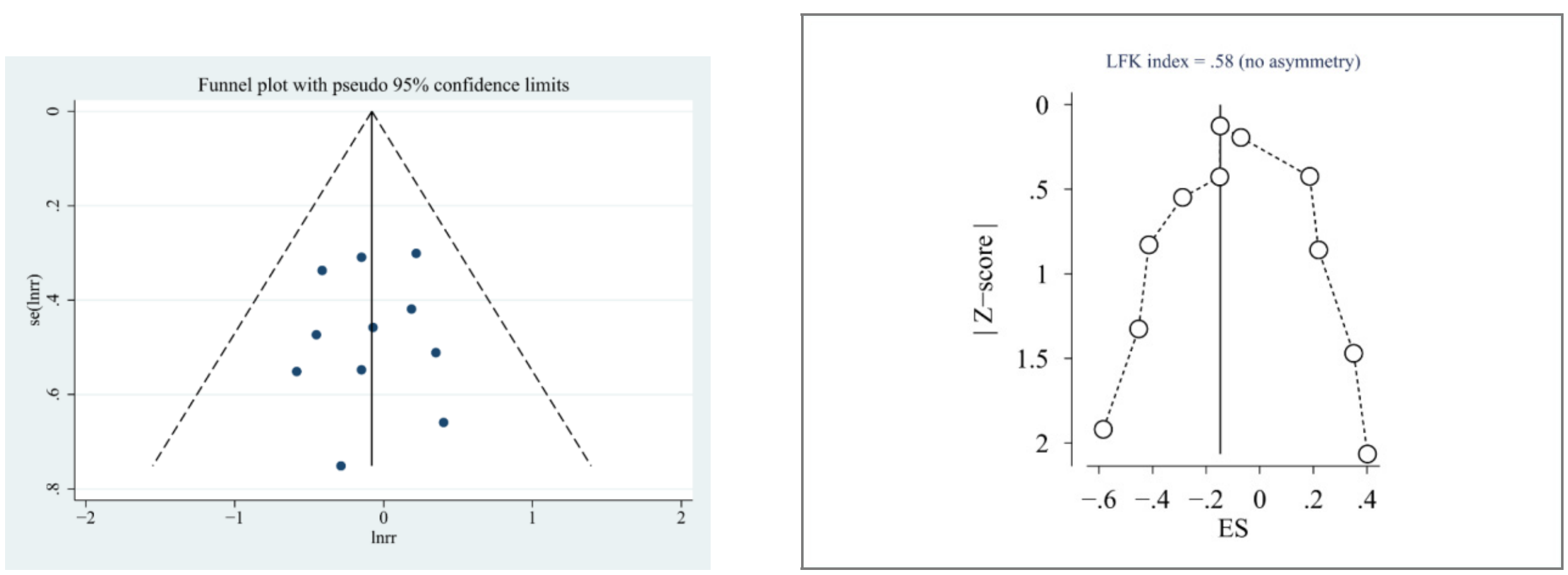

Fig. (S21). Funnel plot and Doi plot of studies evaluating the effect of IDegLira on serious adverse events: (**a)** Funnel plot. (**b)** Doi plot. WMD: weighted mean difference, se: standard error, ES: effect size.

```
. meta trimfill, estimator(linear) funnel eform

  Effect-size label: Log risk-ratio
        Effect size: _meta_es
          Std. err.: _meta_se

Nonparametric trim-and-fill analysis of publication bias
Linear estimator, imputing on the left

Iteration                               Number of studies =     13
  Model: Random-effects                          observed =     10
 Method: REML                                    imputed =      3

Pooling
  Model: Random-effects
 Method: REML
```

Studies	Risk ratio	[95% conf.	interval]
Observed	0.995	0.962	1.029
Observed + Imputed	0.984	0.954	1.016

Fig. (S22). The result of non-parametric trim and fill analysis of the pooled incidence of adverse events using the random-effect analysis.
